# Supplementary material for: Health economic assessment of a scenario to promote bicycling as active transport in Stockholm, Sweden
Source: BMJ Open. 2019 Sep 17;9(9):e030466. doi: 10.1136/bmjopen-2019-030466 (PMC6756337; doi:10.1136/bmjopen-2019-030466)
Supplement: Author's manuscript [file bmjopen-2019-030466.draft_revisions.pdf]

BMJ Open

BMJ Open is committed to open peer review. As part of this commitment we make the peer review history of every article we publish publicly available.

When an article is published we post the peer reviewers' comments and the authors' responses online. We also post the versions of the paper that were used during peer review. These are the versions that the peer review comments apply to.

The versions of the paper that follow are the versions that were submitted during the peer review process. They are not the versions of record or the final published versions. They should not be cited or distributed as the published version of this manuscript.

BMJ Open is an open access journal and the full, final, typeset and author-corrected version of record of the manuscript is available on our site with no access controls, subscription charges or pay-per-view fees (http://bmjopen.bmj.com).

If you have any questions on BMJ Open's open peer review process please email info.bmjopen@bmj.com

Health economic assessment of a scenario to promote bicycling as active transport in Stockholm, Sweden

Journal:	*BMJ Open*
Manuscript ID	bmjopen-2019-030466
Article Type:	Research
Date Submitted by the Author:	15-Mar-2019

Complete List of Authors:	Kriit, Hedi; Umeå Universitet Medicinska fakulteten, Sustainable Health<br>Williams, Jennifer Stewart; Umeå Universitet Medicinska fakulteten, Department of Epidemiology and Global Health<br>Lindholm, Lars; Umeå Universitet Medicinska fakulteten, Department of Epidemiology and Global Health<br>Forsberg, Bertil; Umeå University, Public Health and Clinical Medicine<br>Sommar, Johan; Umea University Department of Public Health and Clinical Medicine, Sustainable health

Keywords:	HEALTH ECONOMICS, DALY, active commuting, Health impacts, Cost-effectiveness analysis, Transport mode shift

SCHOLARONE™
Manuscripts

Health economic assessment of a scenario to promote bicycling as active transport in Stockholm, Sweden

Hedi Katre Kriit[1] Jennifer Stewart Williams[2 3] Lars Lindholm[2] Bertil Forsberg[1] Johan Nilsson Sommar[1]

[1] Section of Sustainable Health
Department of Public Health and Clinical Medicine
Umeå University
SE-90185, Umeå
Sweden

[2] Department of Epidemiology and Global Health
Umeå University
SE-90187, Umeå
Sweden

[3] Research Centre for Generational Health and Ageing
Faculty of Health, University of Newcastle
New Lambton Heights, NSW 2305
Australia

Corresponding and first author:
Hedi Katre Kriit
Email: Hedi.Kriit@umu.se

Key words: **health economic evaluation, commuting, transport mode shift, health impacts, DALY**

Word count: **3623**

iew only

Abstract

Objectives

To conduct a health economic evaluation of a proposed investment in urban bicycle infrastructure in Stockholm County, Sweden.

Design

A cost-effectiveness analysis is undertaken from a health care perspective. Investment costs over a 50 year life cycle are offset by averted health care costs and compared with estimated long term impacts on morbidity, quantified in Disability Adjusted Life Years (DALYs). The results are re-calculated under different assumptions to model the effects of uncertainty.

Setting

The Municipality of Stockholm (population 2.27 million) committed funds for bicycle path infrastructure with the aim of achieving a 15% increase in the number of bicycle commuters by 2030. This work is informed by a previously constructed scenario, in which records from a number of registers were linked and 111,000 residents with capacity to switch from car to bicycle commuting within metropolitan Stockholm, were identified.

Results

Morbidity impacts and health care costs attributed to increased physical activity, change in air pollution exposure and accident risk are quantified under the scenario. The largest reduction in health care costs is attributed to increased physical activity and the second largest to reduced air pollution exposure among the population of Greater Stockholm. The expected net benefit from the investment is 5.1% of the 2017 Stockholm County health care budget, and 3.7% after discounting. The cost-effectiveness analysis estimates a surplus of €9933⁄DALY

For peer review only

averted (2017 values). This is extremely effective in relation to the Swedish threshold value (€53,000 per DALY averted). The results remained robust under varied assumptions pertaining to reduced numbers of additional bicycle commuters.

Conclusion

Investing in urban infrastructure to increase bicycling as active transport is cost-effective from a health care sector perspective.

Article summary

Strengths and limitations

- Health impacts of a transport mode switch from car to bicycle commuting can help avert health sector costs over the long term through improvements in morbidity.
- Offsetting impacts on physical activity, air pollution exposure and accident risk occur within population sub-groups.
- Public investment done into infrastructure is reported to be very cost effective from a health care sector perspective.
- Secondary data was used to estimate the life-time costs of chronic diseases.

For peer review only

Background

Four chronic illnesses – cardiovascular disease, cancer, type 2 diabetes and respiratory disorders - account for at least 60% of global deaths. Several known modifiable lifestyle behaviours such as tobacco smoking, unhealthy diets, harmful alcohol use and physical inactivity impact on these chronic illness through associations with risk factors such as obesity, hypertension and high blood sugar levels [1-6]. Physical inactivity is the fourth most important risk factor for premature mortality in the European Union after tobacco smoking, high blood pressure and overweight [5].

There is now widespread evidence of the importance of physical activity in promoting and maintaining good health [3 5 6]. Even the relatively inactive can benefit from marginal increases in physical activity [7]. Since 2010, when the first official set of global recommendations for physical activity were launched by the World Health Organization (WHO)[3], policies to address insufficient physical activity have been implemented in over half WHO Member States [6].

Currently half of the world's population live in urban environments and this proportion is expected to reach 60% by 2030 [8 9]. Participation in physical activity can be difficult in cities where the heavy use of motorized transportation is estimated to be responsible for 70% of environmental urban air pollution and 13% of global green gas emissions [10]. In 2010 road transport contributed 40% of the economic costs of premature deaths from air pollution in the WHO European Region [11].

Active transport through, for example, bicycling as a way of commuting to work, has been suggested as a way of increasing physical activity and reducing motorised traffic emissions in urban areas. However the health gains for those switching from car to bike transport in heavy traffic areas are offset by the increased risk of serious injury and greater exposure to poor air

For peer review only

quality. Furthermore the distribution of costs and benefits is not equally spread across socio-economic and demographic groups [7 12 13].

In a study of the cost-effectiveness of interventions to encourage more physical activity in the Australian population, Cobiac et al reported that a package of physical activity interventions that include active transport could yield both public health benefits and health sector cost savings [14]. Another study in Australia estimated that health care costs would be reduced by $A1.12 per bicycled kilometer [15]. Studies using previously published disease specific health care costs have estimated significant cost reductions based on hypothetical scenarios of increased active commuting in the United Kingdom [16] and Italy [17]. For example, Jarret et al. estimated that the National Health Service could save roughly £17 billion within 20 years due to reduced prevalence of seven non-communicable diseases (NCDs) resulting from increased walking and bicycling [16]. A number of economic assessments have suggested that travel/transport-related interventions are cost-effective in terms of costs per healthy life-year gained in adult populations [7 18]. Although, not without methodological challenges, there is a need to evaluate the costs and benefits of interventions that promote physical activity through active transport from a health sector perspective [19 20].

In 2015 NCDs accounted for €115 billion in health care costs in European countries [21]. Strategies that address physical inactivity can deliver public health gains through the prevention of chronic diseases. Active transport strategies involving a shift from motor transport can also have wider benefits for the population through improvements in air quality. Sound economic evaluations are needed to build an evidence base in this area [2].

This paper presents a health economic evaluation of a proposed investment in urban bicycle infrastructure in Stockholm, Sweden, from a health care sector perspective. The long term

For peer review only

change in outcomes and health care costs are quantified within a scenario of increased commuting by bicycle and cost-effectiveness is derived in relation to the planned investment.

Methods

Evaluation scenario

As part of its 2018-2030 plan, the Municipality of Stockholm (population 2.27 million) committed funds for bicycle path infrastructure with the aim of achieving a 15% increase in bicycle commuters by 2030. Based on the previously developed transport shift scenario it is estimated that 111, 487 current car drivers could potentially switch to bicycle commuting between home and work. Health impacts, attributed to increased physical activity, differential exposure to air pollution and traffic accidents, and changes in health care costs are evaluated for potential bicycle commuters, current bicycle commuters and the general population, against a fifty year life cycle investment for the bicycle path infrastructure [22].

Population base

The study population is based on a scenario constructed for a previous study in which data on geolocation, demography and commuting modes by origin and destination zone were referenced [22]. Home and workplace addresses were retrieved from the ASTRID database, which has been collecting individual-level data on the Swedish population for over 50 years [23]. Records from ASTRID were matched with the LuTrans transport data base [24] which contains updated information about traffic flow. Travel survey and register data on car ownership were used to allocate individuals to different modes of commuting. Regression functions was used to model the probability for each mode of travel between small statistical areas of Stockholm County. A total of 111, 487 individuals drawn from the population register of Stockholm Municipality were identified as potential bicycle commuters based on geographic distance

For peer review only

from home-to-work and expected bicycle speed based on age and sex. Eligibility required an estimated bicycle commute of 30 minutes or less each way [22 25].

Estimating health outcomes

The estimated health impacts of increased physical activity among potential bicycle commuters are quantified in terms of decreased risk of type 2 diabetes, myocardial infarction (MI), stroke, dementia, colon cancer, breast cancer and heart failure. The change in air pollution exposure is assumed to benefit current bicycle commuters and the general population and disadvantage additional bicycle commuters in terms of risk for asthma, lung cancer, type 2 diabetes, stroke and MI [26]. Health effects of injuries attributable to traffic accidents under this scenario of 111,487 potential bicycle commuters are also estimated [27]. Nilsson Sommar et al [26] estimated changes in morbidity and mortality associated with the above chronic diseases and traffic accidents under the scenario, for potential bicycle commuters, current bicycle commuters, and the general population. Expected cases of disability and premature death were converted to Disability Adjusted Life Years (DALYs) which were calculated using a cohort life table approach [26].

Estimating cost-effectiveness

Information on the budget allocation for the bicycle pathways was obtained from the Stockholm Traffic Planning Office [22 28]. The expected life cycle for new bicycle paths constructed in 2018 is fifty years [29].

The investment period of 2018-2030 was used as a build-up time to reach the estimated amount of additional bicyclists. We estimate that yearly investment costs will vary due to political and other circumstances. The number of additional bicyclists estimated to start active commuting in a given year is a proportion, of the estimated total sample, based on that years

For peer review only

investment allocation (approximately 15 million euros/per year). Health impacts were simultaneously estimated consistent with the proportion of additional bicyclists in a given year. After 2030 an estimated 111,487 additional bicyclists were expected to commute. The number of DALYs attributable to this transport mode change was modelled for the remaining years. Long term health care costs (added and averted) are estimated in accordance with the change in health outcomes attributed to: 1) increased physical activity by potential bicycle commuters; 2) air pollution change for potential bicycle commuters; 3) air pollution change for current bicycle commuters; 4) air pollution change for the general population, and 5) traffic accidents incurred by potential bicycle commuters. These costs are given both in absolute terms and as the proportion of the 2017 Stockholm Municipality health budget [30].

Health outcomes are calculated for the fifty year period 2018-2068 taking the time interval between exposure and realised health benefits into account. It is estimated for physical activity exposure that it takes two years for 50% of the health benefits and six years for 100% of health benefits to be realised for both MI and stroke [31], seventeen and twenty years respectively for 50% and 100% of the health benefits to be realised for dementia, breast and colon cancer [31] and 3.2 and 8 years respectively for 50% and 100% of the health benefits to be realised for type 2 diabetes [32]. The impacts of air pollution and traffic injuries were both assumed to occur during the current year.

This cost-effectiveness analysis adopts the CHEERS guidelines for health economic modelling [33]. Costs are reported in euros (2017) and discounted at an annual rate of 3%. Figure 1 shows the steps undertaken.

For peer review only

Sensitivity analyses

Sensitivity analysis is undertaken for the same investment costs assuming that numbers of potential bicycle commuters are as few as 25% of the estimated sample.

Estimating long term health care costs

The literature was used to inform estimates of long term health care costs for type 2 diabetes, MI, stroke, heart failure, dementia, colon cancer, breast cancer, lung cancer and asthma. (Table 1.) For comparability with Sweden, only papers reporting studies in high-income countries in which health care costs were met by public funds, were sought. Inclusion required that study estimates were based on administrative data recording health care costs matched to clinical diagnoses. The search was conducted in *Google Scholar*, *PubMed* and *Web of Science* between 1995 and 2018. Search terms included the health condition of interest and *lifetime/long term costs.* As no studies were found that specifically reported lifetime health care costs for asthma, MI or cancer outcomes, these estimates are based on one-year average health care costs for asthma, six-year average for MI and five-year disease costs for cancer.

Estimates of the lifetime costs for bicycle related injuries were derived from a study conducted in the Netherlands which used national register and hospital data covering the period 1998 and 2012 [34]. Costs extracted from the literature are inflated by the consumer price index and reported in euros (€) at the 2017 base year.

Results

Investment cost

The net infrastructure cost, excluding communication and maintenance costs, is €101 million. Assuming a fifty year life cycle for the bicycle path, a €2 million annual investment gives an average annual cost of €908 per converter.

For peer review only

Changes in health care costs

The fall in chronic disease cases attributable to increased physical activity among additional bicycle commuters is estimated to save €562 million, or €241 million after discounting (3%) giving an average annual return of €11.2 million. (Figure 2a).

Traffic generated air pollutants were measured in nitrogen oxide concentrations [35]**.** Lower health care costs attributed to decreased air pollution exposure among current bicycle commuters and the general population are estimated to save €1 million annually. This is offset by increased health care costs of €0.28 million for additional bicycle commuters who are assumed to experience increased exposure to air pollution. (Figure 2b).

The annual additional health care costs for injuries sustained as a result of the increased accident risk among additional bicycle commuters is estimated at €1.76 million.

Stockholm Council could avert 5.1 % of its planned 2017 health care expenditure, or 3.7% after discounting [30].

Cost-effectiveness ratio

A cost-effectiveness ratio (CER) is evaluated for the given scenario compared with assumed no change in bicycle commuting. The CER is calculated by subtracting investment costs from total estimated health care savings and dividing by the estimated reduction of DALYs giving a CER of €9933/DALY averted.

Modelling the sensitivity of the estimates

The scenario assumed 111,487 additional bicycle commuters. The sensitivity of the estimates was assessed by assuming fewer potential bicycle commuters. If only 25% of the 111, 487 potential bicycle commuters were to change their mode of transport, the CER would still result in a surplus of €2643/DALY averted. (Figure 3).

For peer review only - http://bmjopen.bmj.com/site/about/guidelines.xhtml

Discussion

This is the first health economic evaluation of this type to use an evidence-based scenario that includes information on geolocation, demography and commuting modes by origin and destination zone drawn from a population register. Investment costs were compared with estimated long term health care costs and savings attributed to increased physical activity, change in air pollution exposure and accident risk. To the best of our knowledge this is also the first economic evaluation to include morbidity health impacts in all three areas of impact. The investment is cost-effective from a health care sector perspective. Estimated net savings are 5.1% of the Stockholm County current health care budget, or 3.7% after discounting.

Physical activity

The largest reduction in health care costs is attributed to increased physical activity. Previous health economic assessments using hypothetical scenarios in Europe have also demonstrated cost savings in the health care sector attributed to physical activity [16 17]. In the United States (US) Grabow et al. [36] showed that, in a population of 2 million across eleven metropolitan areas, if one-half of the commutes, of less than eight kilometer round trip, were made by bicycle instead of car, mortality would be reduced by 700 premature deaths. Based on the US statistical value of life this is equivalent to $US3.8 billion (2010 values) annually or $1900 per individual [36]. In our study the estimated cost saving attributed to reduced morbidity from increased physical activity is €562 million or €100 per annum per additional bicycle commuter.

The benefits from different types of physical acitivity are variable and not necessarily linear. In their systematic review and meta-analysis of 22 cohort studies including over 977,000 people, Woodcock et al [37] concluded that, compared with no activity, two and a half hours per week of moderate intensity activity was associated with a 19% reduction in mortality risk and

For peer review only

seven hours per week of moderate activity reduced mortality risk by 24%. reported in a meta-analysis that the relative risk (RR) would be reduced by 19% if currently inactive person took up vigourous physical activity (2.5hours per week). In this study physical activity effects are reported only for bicycling although adjustments were made for other likely types of physical activity based on individuals' assumed physical capacity adjusted for age and sex.

Air pollution

Some other health economic analyses have assessed the economic value of a reduction in morbidity and mortality due to a reduction in air pollution exposure. Lindsay et al [38] for example, estimated the effect of reduced air pollution exposure on mortality, numbers of active days, and acute cardiac and respiratory hospital admissions, by calculating cost per health event and the statistical value of life based on New Zealand willingness to pay studies. Grabow et al. [36] used a BenMAP cost-benefit regulatory analysis to address the potential benefits attributable to an air pollution reduction in the general population as a result of a transport mode change from car to bicycle for trips of eight kilometers or less in various US cities. Based on acute hospital costs attributable to reduced automobile exhaust and increased ozone exposure net benefits were estimated at $US 4.94 billion (2010 values).

To the best of our knowledge this economic evaluation is also the first to model the impact of a change in air pollution exposure within the general population as well as among current bicycle commuters. Reduced health care costs within the general population (€54 million) are offset by additional costs of €14million among additional bicycle commuters, giving a net freed opportunity cost of €40 million within the health care budget over the 50 year life length of the investment.

For peer review only

Injuries

In their health economic assessment of cycling promotion in Florence, Italy, Taddei et al [17] predicted decreased traffic accidents. Woodcock et al. [39] modelled the health impacts of active transport visions in England and Wales and also predicted a fall in road traffic and accidents under scenarios of increased walking and cycling. However using the same general scenario for increased bicycling in Stockholm County, Nilsson et al. [27] estimated health loss through increased DALYs due to increased numbers and types of traffic accidents being the first major study to highlight the negative impacts of increased injuries and fatalities resulting from the shift from car to bicycle. A strength of the Nilsson study, the results of which are incorported into our analysis of the health impacts and costs of traffic injuries, is the use of nationally representative hospítal and police register data on injuries occured.

Head and spinal injuries costswere included in an analysis of the effects of increased active travel in urban England and Wales on the National Health Service.[16] However these estimates were four times higher than the estimates used here. The current work is based on a study by Scholten et al [34] which assumed traumatic brain injuries occurring in about 9% of bicycle related traffic accidents.

It has been claimed that 60% of the bicycle accidents occur due to poor infrastructure for bicycling, in Sweden [40] and one of the aims of the Stockholm Bicycle Plan was to increase bicycle safety by building infrastructure for active commuters [28]. Improved road infrastructure has been shown to reduce bicycle fatalities by 45% [41] and it has been suggested that increasing the number of bicyclists can also have a “safety in numbers” effect which reduces accident risk [42]. An OECD study [43] showed that Sweden had the world’s safest roads with the lowest

For peer review only

number of fatal accidents per billion-cycled kilometers compared with other European countries.

Total economic benefits

Health care costs

This study shows a net benefit corresponding to 5.1% of the 2017 Stockholm County health care budget. Grabow et al. [36] reported a 5% reduction in health care cost attributable to a change in air pollution exposure and increased physical activity. In a scenario of daily biking of 3.4 kilometers among urban residents in England and Wales, Jarrett et al. [16] reported that the costs for England National Health Service would be reduced by 0.8% as a consequence of increased physical activity.

Economic assessment

It is assumed that investment in physical infrastructure would lead to increased physical activity levels, and flow-on cost reductions within the health care sector. These assumptions have been adopted by others undertaking similar economic evaluations in which investment in infrastructure to promote active transport is assumed to increase physical activity and reduce air pollution. For example, a study conducted in Dane County in the US reported a cost benefit ratio of 1.87 over a ten-year cycle attributed to a $US450 million investment in sidewalks [44]. Some researchers suggest that the saved opportunity costs could finance the investment. For example a study of air quality and exercise-related health benefits resulting from reduced car travel in the Midwestern US Grabow et al.[36] concluded that the estimated reduction in health care costs could finance the infrastructure change within one to ten years. In Italy Taddei et al. [17] estimated that health care costs saved due to a reduction in NCDs could cover 50 kilometers of new bicycle roads within seven to ten years in Florence.

For peer review only

Strengths and limitations

Strengths

Many previous health economic analyses of active transport interventions have used hypothetical or proposed active travel interventions, however the scenario this study is based on was constructed linking several register data that included home and work address coordinates [25]. Gender and age-specific expected bicycle speeds were used to assess individuals' capacity to commute by bicycle between home and work in 30 minutes or less [22 25]. In their systematic review of active transport interventions that include physical activity benefits, Brown et al [45] reported a limited inclusion of health effects with few studies reporting reductions in morbidity as well as mortality. Morbidity reduction is captured in the DALY weights in this study and the impacts of mortality under this scenario have been estimated in a companion paper [26]. Additionally the sensitivity analysis showed that the intervention is highly cost-effective even if only one quarter of the potential bicycle commuters were to take up bicycle commuting.

Limitations

The health impact estimates our economic assessment are based on the Global Burden of Disease [46]. We have not included all possible health impacts. We did not, for example, account for impacts on mental health (e.g. depression) and obesity that are expected to result from increased physical activity [47 48]. A study in the US reported that obesity reductions resulting from active transport could save $90.93 million in health care costs [12].

It is possible that health benefits and cost savings are underestimated in this study due to our not fully accounting for the benefits of reduced obesity and improved well-being that may accrue over time and flow-on to the broader community. These effects would be considered

For peer review only

in an economic evaluation conducted from a societal perspective, which is beyond the scope of the present analysis. There are other potential savings which were not included. For example, it has been shown that people value the time bicycling to work more than the time spent in motorized transport [49]. If a societal perspective had been applied cost-effectiveness may have been higher.

In using secondary data to estimate long term costs for chronic NCDs there was a lack of evidence for cancer and asthma patients. Estimates are therefore based on best guesses from scientific evidence published in the peer-reviewed literature. Although it is common practice for economic evaluations to draw upon evidence from previous studies, comparing evidence from studies with different methodological approaches in different settings is not without its shortcomings [50]. Studies from other high-income countries were used so that the results could be reasonably generalised to Sweden.

Conclusions

From a health care perspective investing in urban infrastructure to increase bicycling in Stockholm is extremely cost-effective in relation to the Swedish threshold value of € 53,000 per DALY averted. This evaluation makes best use of available data and calculates estimates based on a previously developed realistic scenario. More work of this type is needed in order to build an evidence base for decision-makers responsible for investing funds in public infrastructure.

Author Contributions

HKK planned the health economic study and undertook the data collection and economic analysis with input from LL. HKK wrote the first draft. JSW reviewed the overall content, provided input and revised and re-drafted the paper in conjunction with HKK. BF advised on

For peer review only

the planning and conduct of the study and was responsible for the overall transport scenario project of which this work was part. JNS provided health impact estimates and critical input into the planning and conduct of the study, assisted with the statistical analyses, and helped revise the drafts. All authors read and approved the final version of the manuscript.

Role of the funding source

This work was funded by the Swedish Research Council for Health, Working Life and Welfare (grant number 2012-1296). The paper reflect only the authors views, and the funder had no role in the design or conduct of the study.

Competing interests

All authors have completed the ICMJE uniform disclosure form at www.icmje.org/coi_disclosure.pdf and declare: no support from any organization for the submitted work; no financial relationships with any organizations that might have an interest in the submitted work in the previous three years; no other relationships or activities that could appear to have influenced the submitted work.

Transparency declaration

The lead author affirms that the manuscript is an honest, accurate, and transparent account of the study being reported; that no important aspects of the study have been omitted; and that any discrepancies from the study as planned have been explained.

Patient and Public involvement: No patient or public involvement.

The datasets analyzed during the current study are available from the corresponding author on reasonable request.

Acknowledgements

Bertil Forsberg received a grant (2012-1296) from FORTE (Forskningsrådet för hälsa, arbetsliv och välfärd) for the transport scenario study.

For peer review only

We thank study leaders and co-authors in the transport scenario study, in particular Peter Schantz, Christer Johansson, Wasif Raza and Magnus Strömgren.

For peer review only

References

1. World Health Organization. 2008-2013 Action Plan for the Global Strategy for the Prevention and Control of Noncommunicable Diseases. Genève, 2008:48.

2. Cecchini M, Sassi F, Laver JA, et al. Tackling of unhealthy diets, physical inactivity, and obesity: health effects and cost-effectiveness. *The Lancet* 2010;376:1775-84. doi: DOI:10.1016/S0140-6736(10)61514-0

3. World Health Organization. Global recommendations on physical activity for health. Genève, 2010:60.

4. Lee I-M, Shiroma EJ, Lobelo F, et al. Effect of physical inactivity on major non-communicable diseases worldwide: an analysis of burden of disease and life expectancy. *The Lancet* 2012;380:219-29. doi: http://dx.doi.org/10.1016/50140-6736(12)61031-9

5. World Health Organization, OECD, European Observatory. Promoting health, preventing disease. The economic case. In: Davis McDaid FS, Sherry Merkur, ed. New York, 2015:370.

6. World Health Organization. Physical Activity Fact Sheet. 2016

7. Cecchini M, Bull F. Promoting physical activity. In: David McDaid FS, Sherry Merkur, ed. Promoting health, preventing disease The economic case. New York: McGraw Hill Education 2015:101-34.

8. World Health Organization. Urbanisation and Health. *Bull World Health Organ* 2010;88:245-46.

9. United Nations. Sustainable Development Goals. 2015

10. IPCC. Climate change 2007 - Mitigation of climate change. 2007

11. World Health Organization Regional Office for Europe, OECD. Economic cost of the health impact of air pollution in Europe: clean air, health and wealth. Copenhagen, 2015:66.

12. Pucher J, Buehler R, Bassett DR, et al. Walking and cycling to health: a comparative analysis of city, state, and international data. *Am J Public Health* 2010;100(10):1986-92. doi: 10.2105/ajph.2009.189324 [published Online First: 2010/08/21]

13. Fraser SD, Lock K. Cycling for transport and public health: a systematic review of the effect of the environment on cycling. *Eur J Public Health* 2011;21(6):738-43. doi: 10.1093/eurpub/ckq145 [published Online First: 2010/10/12]

14. Cobiac LJ, Vos T, Barendregt JJ. Cost-effectiveness of interventions to promote physical activity: a modelling study. *PLoS Med* 2009;6(7):e1000110. doi: 10.1371/journal.pmed.1000110 [published Online First: 2009/07/15]

15. Mulley C, Tyson R, McCue P, et al. Valuing active travel: including the health benefits of sustainable transport in transportation appraisal frameworks. *Research in Transportation Business and Management* 2013;7:27-34.

16. Jarrett J, Woodcock J, Griffiths UK, et al. Effect of increasing active travel in urban England and Wales on costs to the National Health Service. *The Lancet* 2012;379(9832):2198-205. doi: 10.1016/s0140-6736(12)60766-1 [published Online First: 2012/06/12]

17. Taddei C, Gnesotto R, Forni S, et al. Cycling promotion and non-communicable disease prevention: health impact assessment and economic evaluation of cycling to work or school in Florence. *PLoS One* 2015;10(4):e0125491. doi: 10.1371/journal.pone.0125491 [published Online First: 2015/05/01]

For peer review only - http://bmjopen.bmj.com/site/about/guidelines.xhtml

18. Beale SJ, Bending MW, Trueman P, et al. Should we invest in environmental interventions to encourage physical activity in England? An economic appraisal. *Eur J Public Health* 2012;22(6):869-73. doi: https://doi.org/10.1093/eurpub/ckr151

19. Hanson S, Jones A. Missed opportunities in the evaluation of public health interventions: a case study of physical activity programmes. *BMC Public Health* 2017;17(1):674. doi: 10.1186/s12889-017-4683-z [published Online First: 2017/08/24]

20. Weatherly H, Drummond M, Claxton K, et al. Methods for assessing the cost-effectiveness of public health interventions: key challenges and recommendations. *Health Policy* 2009;93(2-3):85-92. doi: 10.1016/j.healthpol.2009.07.012 [published Online First: 2009/08/28]

21. OECD/EU. Health at a Glance: Europe 2016 - State of Health in the EU Cycle. Paris, 2016:204.

22. Strömgren M, Schantz. P, Nilsson Sommar J, et al. Modelling a commuter modal shift from car trips to cycling: Scenario construction and outcomes for Stockholm County, Sweden. To be submitted., 2019.

23. Stjenström O. The Astrid data base and population geography (only in Swedish "Databasen ASTRID och befolkningsgeografi exemplen integration och barnfamiljenas geografi" *Geografiska Notised*;60. No.2

24. Jonsson D BS, Almström P, Algers S. The usefulness of transport models in Swedish planning practice. *Transport Reviews* 2011;31(2):251-65.

25. Nilsson Sommar J, Schantz P, Strömgren M, et al. The potential for reduced mortality by increased cycle commuting - a health impact assessment using register data on home and work addresses in Stockholm, Sweden. To be submitted., 2019.

26. Nilsson Sommar J, Johansson C, Lövenheim B, et al. Overall health impacts of a potential increase in active commuting in Stockholm, Sweden. To be submitted., 2019.

27. Nilsson P, Stigson H, Ohlin M, et al. Modelling the effect on injuries and fatalities when changing mode of transport from car to bicycle. *Accid Anal Prev* 2017;100:30-36. doi: 10.1016/j.aap.2016.12.020 [published Online First: 2017/01/14]

28. En el av Framkomlighetsstrategin: Cyckelplan. In: Stad S, ed., 2016.

29. Livslängs och Trafikanalys. In: Trafikverket, ed., 2002:6.

30. Stockholm Läns Lansting Hälsa- och sjukvårdsnämden. Slutlig budget 2017 HSN: Förändringsarbete i fokus när vården i länet växer, 2017.

31. Kahlmeier S CN DH, et al. . Health economic assessment tools (HEAT) for walking and cycling: methodology and user guide. *Copenhagen: WHO Regional Office Europe* 2011

32. Umpierre D, Ribeiro PA, Kramer CK, et al. Physical activity advice only or structured exercise training and association with HbA1c levels in type 2 diabetes: a systematic review and meta-analysis. *JAMA* 2011;305(17):1790-9. doi: 10.1001/jama.2011.576 [published Online First: 2011/05/05]

33. Husereau D, Drummond M, Petrou S, et al. Consolidated Health Economic Evaluation Reporting Standards (CHEERS)--explanation and elaboration: a report of the ISPOR Health Economic Evaluation Publication Guidelines Good Reporting Practices Task Force. *Value Health* 2013;16(2):231-50. doi: 10.1016/j.jval.2013.02.002 [published Online First: 2013/03/30]

34. Scholten AC, Polinder S, Panneman MJ, et al. Incidence and costs of bicycle-related traumatic brain injuries in the Netherlands. *Accid Anal Prev* 2015;81:51-60. doi: 10.1016/j.aap.2015.04.022 [published Online First: 2015/05/06]

For peer review only - http://bmjopen.bmj.com/site/about/guidelines.xhtml

35. Johansson C, Lovenheim B, Schantz P, et al. Impacts on air pollution and health by changing commuting from car to bicycle. *Sci Total Environ* 2017;584-585:55-63. doi: 10.1016/j.scitotenv.2017.01.145 [published Online First: 2017/01/31]

36. Grabow ML, Spak SN, Holloway T, et al. Air quality and exercise-related health benefits from reduced car travel in the midwestern United States. *Environ Health Perspect* 2012;120(1):68-76. doi: 10.1289/ehp.1103440 [published Online First: 2011/11/04]

37. Woodcock J, Franco OH, Orsini N, et al. Non-vigorous physical activity and all-cause mortality: systematic review and meta-analysis of cohort studies. *Int J Epidemiology* 2011;40(1):121-38. doi: 10.1093/ije/dyq104 [published Online First: 2010/07/16]

38. Lindsay G, Macmillan A, Woodward A. Moving urban trips from cars to bicycles: impact on health and emissions. *Australian and New Zealand Journal of Public Health* 2011;35(1):54-60. doi: 10.1111/j.1753-6405.2010.00621.x [published Online First: 2011/02/09]

39. Woodcock J, Givoni M, Morgan AS. Health impact modelling of active travel visions for England and Wales using an Integrated Transport and Health Impact Modelling Tool (ITHIM). *PLoS One* 2013;8(1):e51462. doi: 10.1371/journal.pone.0051462 [published Online First: 2013/01/18]

40. Niska A G, S, Nyberg, J, Eriksson, J.Cyklisters singelolyckor - Analys av olycks- och skadedata samt djupintervjuer (Single bicycle accidents analysis of hospital injury data and interviews). *VTI, Linköping, Sweden* 2013

41. Schepers PM, Rob; Wegman, Fred; Heinen, Eva, . Road safety and bicycle usage impacts of unbundling vehicular and cycle traffic in Dutch urban networks. *European Journal of Transport and Infrastructure Research* 2013;13(3):221-38.

42. Jacobsen PL. Safety in numbers: more walkers and bicyclists, safer walking and bicycling. *Inj Prev* 2015;21(4):271-5. doi: 10.1136/ip.9.3.205rep [published Online First: 2015/07/24]

43. OECD/EU. Road Safety Annual Report 2011. OECD International Transport Forum, Paris, 2012.

44. Guo JY, Gandavarapu S. An economic evaluation of health-promotive built environment changes. *Prev Med* 2010;50 Suppl 1:S44-9. doi: 10.1016/j.ypmed.2009.08.019 [published Online First: 2009/10/21]

45. Brown V, Diomedi, B., Moodie, M., Veerman, J., & Carter, R. A systematic review of economic analyses of active transport interventions that include physical activity benefits. *Transport Policy* 2016;45(C):190-208. doi: 10.1016/j.tranpol.2015.10.003

46. World Health Organization. Global burden of disease 2004 update: disability weights for diseases and conditions. Genève: WHO, 2004:9.

47. Gill JM, Malkova D. Physical activity, fitness and cardiovascular disease risk in adults: interactions with insulin resistance and obesity. *Clin Sci* 2006;110(4):409-25. doi: 10.1042/cs20050207 [published Online First: 2006/03/11]

48. Warburton DE, Nicol CW, Bredin SS. Health benefits of physical activity: the evidence. *CMAJ : Canadian Medical Association Journal* 2006;174(6):801-9. doi: 10.1503/cmaj.051351 [published Online First: 2006/03/15]

49. Borjesson M EJ. The value of time and external benefits in bicycle appraisal. *Transport Research Part A Policy and Practice* 2012;46(4):673-83.

50. Briggs A CC, Sculpher M. Decision Modelling for Health Economic Evaluation. Oxford University Press, 2011.

For peer review only - http://bmjopen.bmj.com/site/about/guidelines.xhtml

51. Zhuo X, Zhang P, Hoerger TJ. Lifetime direct medical costs of treating type 2 diabetes and diabetic complications. *Am J Prev Med* 2013;45(3):253-61. doi: 10.1016/j.amepre.2013.04.017 [published Online First: 2013/08/21]

52. Ghatnekar O, Persson U, Asplund K, et al. Costs for stroke in Sweden 2009 and developments since 1997. *Int J Technol Assess Health Care* 2014;30(2):203-9. doi: 10.1017/s0266462314000075 [published Online First: 2014/06/05]

53. Yang Z, Zhang K, Lin PJ, et al. A longitudinal analysis of the lifetime cost of dementia. *Health Serv Res* 2012;47(4):1660-78. doi: 10.1111/j.1475-6773.2011.01365.x [published Online First: 2011/12/17]

54. Tran DT, Ohinmaa AX, Thanh NC, et al. Resource use and burden of hospitalization, outpatient, physician, and drug costs in short - and long-term care after acute myocardial infarction. *Canadian Journal of Cardiology* 2018;34(10):1298-306.

55. Dunlay SM, Shah ND, Shi Q, et al. Lifetime costs of medical care after heart failure diagnosis. *Circ Cardiovasc Qual Outcomes* 2011;4(1):68-75. doi: 10.1161/circoutcomes.110.957225 [published Online First: 2010/12/09]

56. Yabroff KR, Lamont EB, Mariotto A, et al. Cost of care for elderly cancer patients in the United States. *J Natl Cancer Inst* 2008;100(9):630-41. doi: 10.1093/jnci/djn103 [published Online First: 2008/05/01]

57. Jacob C, Bechtel B, Engel S, et al. Healthcare costs and resource utilization of asthma in Germany: a claims data analysis. *Eur J Health Econ* 2016;17(2):195-201. doi: 10.1007/s10198-015-0671-3 [published Online First: 2015/02/27]

For peer review only

For peer review only - http://bmjopen.bmj.com/site/about/guidelines.xhtml

Table 1. Chronic disease treatment costs per case derived from the literature and used to calculate health care costs

Disease	Sample size	Sample population	Data collection	Perspective	Method	Costs per case*[1]	Disease duration	Reference
Type 2 Diabetes	1000	Newly diagnosed type 2 diabetes	2009-2010	Health care	Markov modelling	83 155	Life-time	Zhuo et al. [51]
Stroke	9064	All first stoke cases registered in the national register	2009	Health and societal	Aggregated costs	74 125	Life-time	Ghatnekar et. al [52]
Dementia	111 811	Medicare beneficiaries ≥65years	1997-2005	Health care	Cohort-based simulations	13 765	Life-time	Yang et.al. [53]
Myocardial infarction	41 210	Acute myocardial infarction incidences in Alberta, Canada	2004-2014	Health care	Cohort based aggregated costs	147 262	6-years	Tran et al. [54]

[1] * Costs per case expressed in € 2017 value

For peer review only

Heart failure	1054	Olmstead county residents who fulfilled the special criteria's established for heart failure.	2006	Health care	Tiangand-Huang method	113 975	Life-time	Dunlay et.al, [55]
Breast cancer	77009	Cohort of elderly Medicare, newly diagnosed cancer patients	1998-2004	Health care	5-year aggregated costs	21 177	5-years	Yabroff et.al [56]
Colon cancer	44838	Cohort of elderly Medicare, newly diagnosed cancer patients	1998-2004	Health care	5-year aggregated costs	42, 446	5- years	Yabroff et.al, [56]
Lung cancer	54665	Cohort of elderly Medicare, newly diagnosed cancer patients	1998-2004	Health care	5-year aggregated costs	43, 211	5-years	Yabroff et.al, [56]
Asthma	49 668	Patients with ICD-10-GM asthma code	2010	Health care	Aggregated costs	49 528 [2]	1-year	Jacob et.al,[57]

[2] Multiplied by the average length lived with the disease

For peer review only - http://bmjopen.bmj.com/site/about/guidelines.xhtml

Control group: 248 340	extracted from German sickness fund.

Figure 1. Flowchart for estimated chain of events as the base for conducting the cost-effectiveness analysis

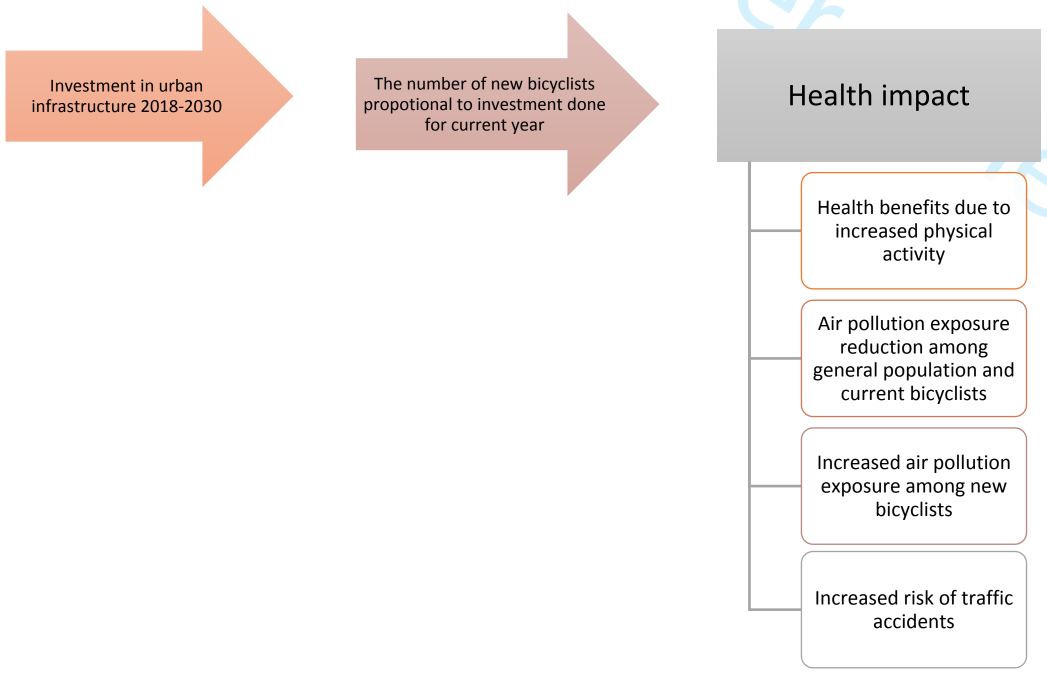

For peer review only

Figure 2a. Estimated yearly expenditure averted (in millions €) in the health care sector due to increased physical activity, air pollution exposure change, and risk of traffic accidents.

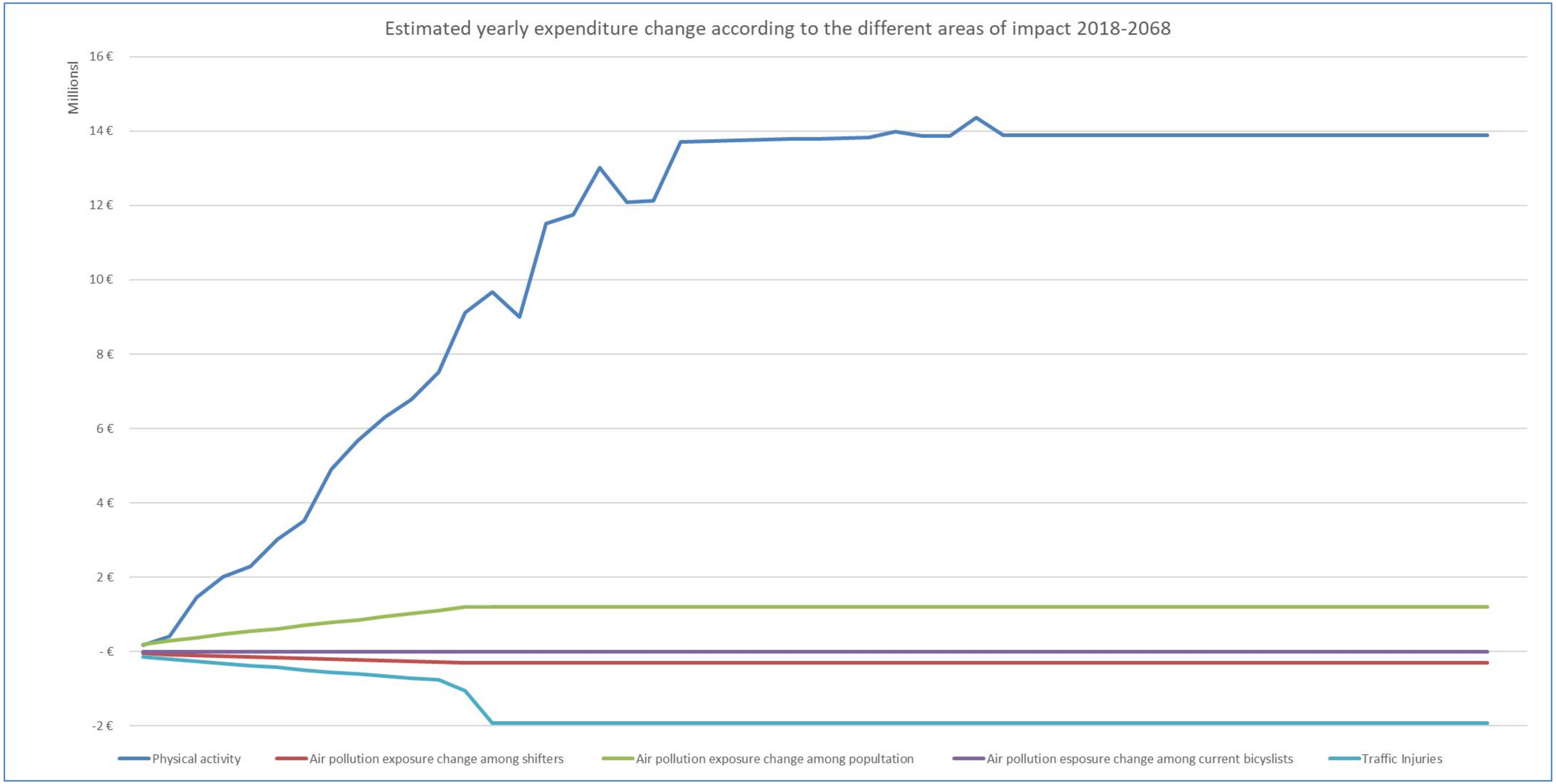

Figure 2b. Estimated average yearly expenditure averted (in millions €) in the health care sector due to increased physical activity, air pollution exposure change and increased risk of traffic accidents

For peer review only

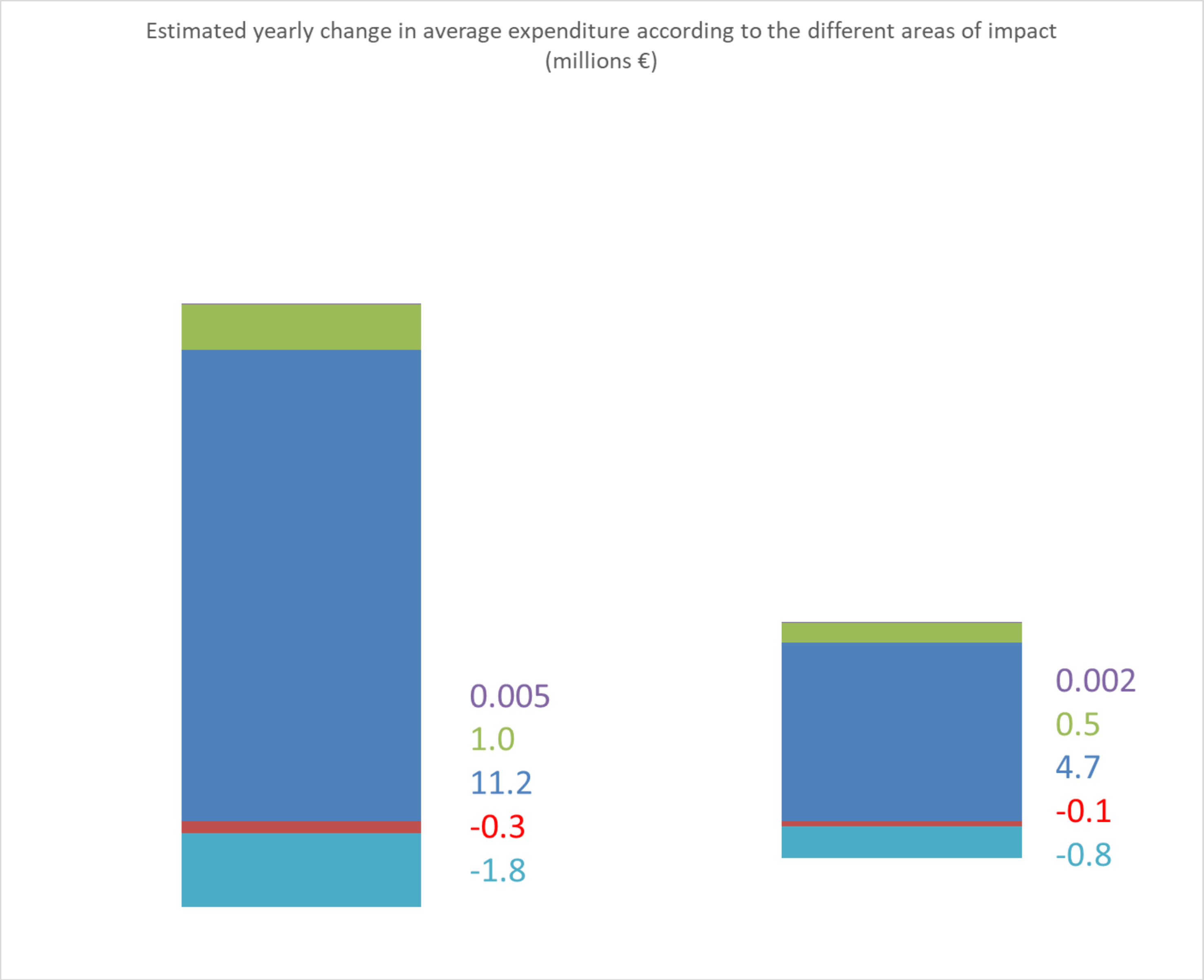
Estimated yearly change in average expenditure according to the different areas of impact (millions €)
0.005
1.0
11.2
-0.3
-1.8
0.002
0.5
4.7
-0.1
-0.8

Figure 3. CER change for percentages of the expected increase in number of bicyclists

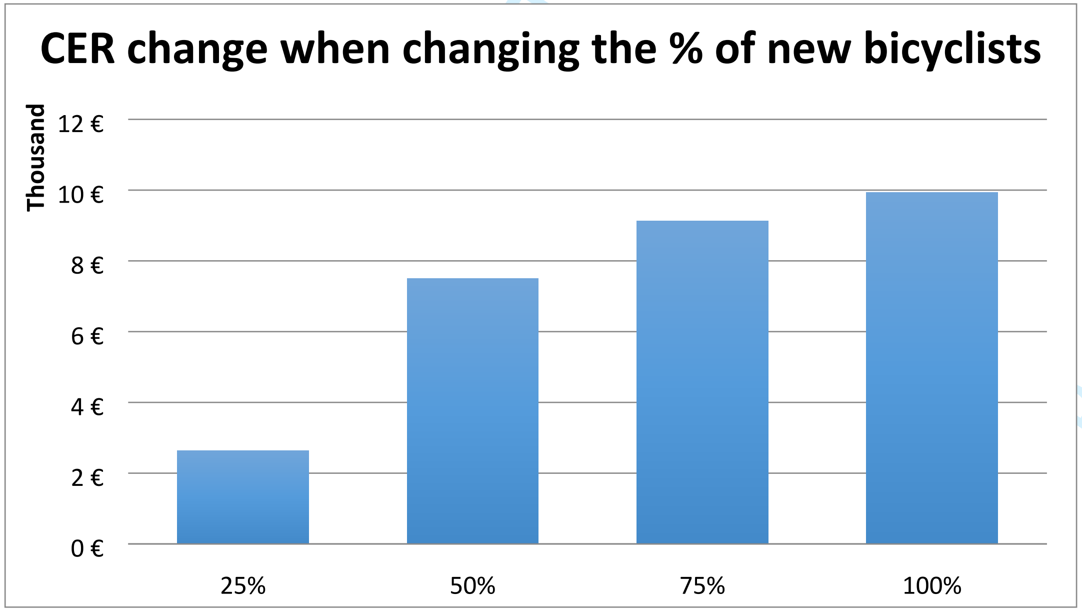

CHEERS Checklist
Items to include when reporting economic evaluations of health interventions

The **ISPOR CHEERS Task Force Report**, *Consolidated Health Economic Evaluation Reporting Standards (CHEERS)—Explanation and Elaboration: A Report of the ISPOR Health Economic Evaluations Publication Guidelines Good Reporting Practices Task Force,* provides examples and further discussion of the 24-item CHEERS Checklist and the CHEERS Statement. It may be accessed via the *Value in Health* or via the ISPOR Health Economic Evaluation Publication Guidelines – CHEERS: Good Reporting Practices webpage: http://www.ispor.org/TaskForces/EconomicPubGuidelines.asp

**Section/item**	**Item No**	**Recommendation**	**Reported on page No/ line No**
**Title and abstract**			
Title	1	Identify the study as an economic evaluation or use more specific terms such as "cost-effectiveness analysis", and describe the interventions compared.	1
Abstract	2	Provide a structured summary of objectives, perspective, setting, methods (including study design and inputs), results (including base case and uncertainty analyses), and conclusions.	2-3
**Introduction**			
Background and objectives	3	Provide an explicit statement of the broader context for the study. Present the study question and its relevance for health policy or practice decisions.	5-6/22
**Methods**			
Target population and subgroups	4	Describe characteristics of the base case population and subgroups analysed, including why they were chosen.	6/14
Setting and location	5	State relevant aspects of the system(s) in which the decision(s) need(s) to be made.	NA
Study perspective	6	Describe the perspective of the study and relate this to the costs being evaluated.	6/5
Comparators	7	Describe the interventions or strategies being compared and state why they were chosen.	NA
Time horizon	8	State the time horizon(s) over which costs and consequences are being evaluated and say why appropriate.	6/11
Discount rate	9	Report the choice of discount rate(s) used for costs and outcomes and say why appropriate.	8/12
Choice of health outcomes	10	Describe what outcomes were used as the measure(s) of benefit in the evaluation and their relevance for the type of analysis performed.	7/20
Measurement of effectiveness	11a	*Single study-based estimates:* Describe fully the design features of the single effectiveness study and why the single study was a sufficient source of clinical effectiveness data.	NA

For peer review only

	11b	*Synthesis-based estimates:* Describe fully the methods used for identification of included studies and synthesis of clinical effectiveness data.	8/18
Measurement and valuation of preference based outcomes	12	If applicable, describe the population and methods used to elicit preferences for outcomes.	7/12
Estimating resources and costs	13a	*Single study-based economic evaluation:* Describe approaches used to estimate resource use associated with the alternative interventions. Describe primary or secondary research methods for valuing each resource item in terms of its unit cost. Describe any adjustments made to approximate to opportunity costs.	NA
	13b	*Model-based economic evaluation:* Describe approaches and data sources used to estimate resource use associated with model health states. Describe primary or secondary research methods for valuing each resource item in terms of its unit cost. Describe any adjustments made to approximate to opportunity costs.	7-8/17
Currency, price date, and conversion	14	Report the dates of the estimated resource quantities and unit costs. Describe methods for adjusting estimated unit costs to the year of reported costs if necessary. Describe methods for converting costs into a common currency base and the exchange rate.	8/12
Choice of model	15	Describe and give reasons for the specific type of decision-analytical model used. Providing a figure to show model structure is strongly recommended.	7/Figure 1
Assumptions	16	Describe all structural or other assumptions underpinning the decision-analytical model.	7/20
Analytical methods	17	Describe all analytical methods supporting the evaluation. This could include methods for dealing with skewed, missing, or censored data; extrapolation methods; methods for pooling data; approaches to validate or make adjustments (such as half cycle corrections) to a model; and methods for handling population heterogeneity and uncertainty.	8/20
**Results**			
Study parameters	18	Report the values, ranges, references, and, if used, probability distributions for all parameters. Report reasons or sources for distributions used to represent uncertainty where appropriate. Providing a table to show the input values is strongly recommended.	Tabel 1. 9/18
Incremental costs and outcomes	19	For each intervention, report mean values for the main categories of estimated costs and outcomes of interest, as well as mean differences between the comparator groups. If applicable, report incremental cost-effectiveness ratios.	10/18
Characterising uncertainty	20a	*Single study-based economic evaluation:* Describe the effects of sampling uncertainty for the estimated incremental cost and incremental effectiveness parameters, together with the impact	NA

For peer review only

		of methodological assumptions (such as discount rate, study perspective).	
	20b	*Model-based economic evaluation:* Describe the effects on the results of uncertainty for all input parameters, and uncertainty related to the structure of the model and assumptions.	10/20
Characterising heterogeneity	21	If applicable, report differences in costs, outcomes, or cost-effectiveness that can be explained by variations between subgroups of patients with different baseline characteristics or other observed variability in effects that are not reducible by more information.	NA
**Discussion**			
Study findings, limitations, generalisability, and current knowledge	22	Summarise key study findings and describe how they support the conclusions reached. Discuss limitations and the generalisability of the findings and how the findings fit with current knowledge.	11/18; 15/16
**Other**			
Source of funding	23	Describe how the study was funded and the role of the funder in the identification, design, conduct, and reporting of the analysis. Describe other non-monetary sources of support.	16/5
Conflicts of interest	24	Describe any potential for conflict of interest of study contributors in accordance with journal policy. In the absence of a journal policy, we recommend authors comply with International Committee of Medical Journal Editors recommendations.	

For consistency, the CHEERS Statement checklist format is based on the format of the CONSORT statement checklist

The **ISPOR CHEERS Task Force Report** provides examples and further discussion of the 24-item CHEERS Checklist and the CHEERS Statement. It may be accessed via the *Value in Health* link or via the ISPOR Health Economic Evaluation Publication Guidelines – CHEERS: Good Reporting Practices webpage: http://www.ispor.org/TaskForces/EconomicPubGuidelines.asp

The citation for the CHEERS Task Force Report is:
Husereau D, Drummond M, Petrou S, et al. Consolidated health economic evaluation reporting standards (CHEERS)—Explanation and elaboration: A report of the ISPOR health economic evaluations publication guidelines good reporting practices task force. Value Health 2013;16:231-50.

For peer review only

BMJ Open

Health economic assessment of a scenario to promote bicycling as active transport in Stockholm, Sweden

Journal:	*BMJ Open*
Manuscript ID	bmjopen-2019-030466.R1
Article Type:	Research
Date Submitted by the Author:	05-Jun-2019

Complete List of Authors:	Kriit, Hedi; Umeå Universitet Medicinska fakulteten, Sustainable Health<br>Williams, Jennifer Stewart; Umeå Universitet Medicinska fakulteten, Department of Epidemiology and Global Health<br>Lindholm, Lars; Umeå Universitet Medicinska fakulteten, Department of Epidemiology and Global Health<br>Forsberg, Bertil; Umeå University, Public Health and Clinical Medicine<br>Sommar, Johan; Umea University Department of Public Health and Clinical Medicine, Sustainable health

**Primary Subject Heading**:	Health economics
Secondary Subject Heading:	Health economics, Health policy, Public health
Keywords:	HEALTH ECONOMICS, DALY, active commuting, Health impacts, Cost-effectiveness analysis, Transport mode shift

SCHOLARONE™
Manuscripts

Health economic assessment of a scenario to promote bicycling as active transport in Stockholm, Sweden

Hedi Katre Kriit[1] Jennifer Stewart Williams[2 3] Lars Lindholm[2] Bertil Forsberg[1] Johan Nilsson Sommar[1]

[1] Section of Sustainable Health
Department of Public Health and Clinical Medicine
Umeå University
SE-90185, Umeå
Sweden

[2] Department of Epidemiology and Global Health
Umeå University
SE-90187, Umeå
Sweden

[3] Research Centre for Generational Health and Ageing
Faculty of Health, University of Newcastle
New Lambton Heights, NSW 2305
Australia

Corresponding and first author:
Hedi Katre Kriit
Email: Hedi.Kriit@umu.se

iew only

Key words: **health economic evaluation, commuting, transport mode shift, health impacts, DALY**

Word count: **3862**

For peer review only - http://bmjopen.bmj.com/site/about/guidelines.xhtml

Abstract

Objectives

To conduct a health economic evaluation of a proposed investment in urban bicycle infrastructure in Stockholm County, Sweden.

Design

A cost-effectiveness analysis is undertaken from a health care perspective. Investment costs over a 50 year life cycle are offset by averted health care costs and compared with estimated long term impacts on morbidity, quantified in Disability Adjusted Life Years (DALYs). The results are re-calculated under different assumptions to model the effects of uncertainty.

Setting

The Municipality of Stockholm (population 2.27 million) committed funds for bicycle path infrastructure with the aim of achieving a 15% increase in the number of bicycle commuters by 2030. This work is based on a previously constructed scenario, in which individual registry data on home and work address and a transport modelled allocation to different modes of transport identified 111,000 individuals with the physical capacity to bicycle to work within 30 minutes but that currently drive a car to work.

Results

Morbidity impacts and health care costs attributed to increased physical activity, change in air pollution exposure and accident risk are quantified under the scenario. The largest reduction in health care costs is attributed to increased physical activity and the second largest to reduced air pollution exposure among the population of Greater Stockholm. The expected net benefit from the investment is 5.1% of the 2017 Stockholm County health care budget, and

For peer review only - http://bmjopen.bmj.com/site/about/guidelines.xhtml

For peer review only

3.7% after discounting. The cost-effectiveness analysis estimates a surplus of €9933⁄DALY averted (2017 values). This is extremely effective in relation to the Swedish threshold value (€53,000 per DALY averted). The results remained robust under varied assumptions pertaining to reduced numbers of additional bicycle commuters.

Conclusion

Investing in urban infrastructure to increase bicycling as active transport is cost-effective from a health care sector perspective.

Article summary

Strengths and limitations

- Health impacts of a transport mode switch from car to bicycle commuting can help avert health sector costs over the long term through improvements in morbidity.
- Offsetting impacts on physical activity, air pollution exposure and accident risk occur within population sub-groups.
- Public investment done into infrastructure is reported to be very cost-effective from a health care sector perspective.
- Secondary data was used to estimate the life-time costs of chronic diseases.

For peer review only

Background

Four chronic illnesses – cardiovascular disease, cancer, type 2 diabetes and respiratory disorders - account for at least 60% of global deaths. Several known modifiable lifestyle behaviours such as tobacco smoking, unhealthy diets, harmful alcohol use and physical inactivity impact on these chronic illness through associations with risk factors such as obesity, hypertension and high blood sugar levels [1-6]. Physical inactivity is the fourth most important risk factor for premature mortality in the European Union after tobacco smoking, high blood pressure and overweight [5].

There is now widespread evidence of the importance of physical activity in promoting and maintaining good health [3 5 6]. Even the relatively inactive can benefit from marginal increases in physical activity [7]. Since 2010, when the first official set of global recommendations for physical activity were launched by the World Health Organization (WHO)[3], policies to address insufficient physical activity have been implemented in over half WHO Member States [6].

Currently half of the world's population live in urban environments and this proportion is expected to reach 60% by 2030 [8 9]. Participation in physical activity can be difficult in cities where the heavy use of motorized transportation is estimated to be responsible for 70% of environmental urban air pollution and 13% of global green gas emissions [10]. In 2010 road transport contributed 40% of the economic costs of premature deaths from air pollution in the WHO European Region [11].

Active transport through, for example, bicycling as a way of commuting to work, has been suggested as a way of increasing physical activity and reducing motorised traffic emissions in urban areas. However the health gains for those switching from car to bike transport in heavy traffic areas are offset by the increased risk of serious injury and greater exposure to poor air

For peer review only - http://bmjopen.bmj.com/site/about/guidelines.xhtml

quality. Furthermore the distribution of costs and benefits is not equally spread across socio-economic and demographic groups [7 12 13].

In a study of the cost-effectiveness of interventions to encourage more physical activity in the Australian population, Cobiac et al reported that a package of physical activity interventions that include active transport could yield both public health benefits and health sector cost savings [14]. Another study in Australia estimated that health care costs would be reduced by $A1.12 per bicycled kilometer [15]. Studies using previously published disease specific health care costs have estimated significant cost reductions based on hypothetical scenarios of increased active commuting in the United Kingdom [16] and Italy [17]. For example, Jarret et al. estimated that the National Health Service could save roughly £17 billion within 20 years due to reduced prevalence of seven non-communicable diseases (NCDs) resulting from increased walking and bicycling [16]. A number of economic assessments have suggested that travel/transport-related interventions are cost-effective in terms of costs per healthy life-year gained in adult populations [7 18]. Although, not without methodological challenges, there is a need to evaluate the costs and benefits of interventions that promote physical activity through active transport from a health sector perspective [19 20].

In 2015 NCDs accounted for €115 billion in health care costs in European countries [21]. Strategies that address physical inactivity can deliver public health gains through the prevention of chronic diseases. Active transport strategies involving a shift from motor transport can also have wider benefits for the population through improvements in air quality. Sound economic evaluations are needed to build an evidence base in this area [2].

This paper presents a health economic evaluation of a proposed investment in urban bicycle infrastructure in Stockholm, Sweden, from a health care sector perspective. The long term

For peer review only - http://bmjopen.bmj.com/site/about/guidelines.xhtml

change in outcomes and health care costs are quantified within a scenario of increased commuting by bicycle and cost-effectiveness is derived in relation to the planned investment.

Methods

Evaluation scenario

As part of its 2018-2030 plan, the Municipality of Stockholm (population 2.27 million) committed funds for bicycle path infrastructure with the aim of achieving a 15% increase in bicycle commuters by 2030. Based on the previously developed transport shift scenario it is estimated that 111, 487 current car drivers could potentially switch to bicycle commuting between home and work. Thereafter, in the economic evaluation it is assumed that yearly investment costs lead to a proportional amount of new bicyclists from the estimated number of potential converters. Health impacts, attributed to increased physical activity, differential exposure to air pollution and traffic accidents, and changes in health care costs are evaluated for potential bicycle commuters, current bicycle commuters and the general population. The timeline assumes that it takes 12 years of investment for all estimated potential bicyclists to shift their mode of transport and health impacts are calculated for a fifty year life length of the investment in bicycle path infrastructure [22]. Yearly health impacts are compared, ceteris paribus, against the current 2017 situation and health care budget.

Population base

The study population is based on a scenario constructed for a previous study in which data on geolocation, demography and commuting modes by origin and destination zone were referenced [22]. Home and workplace addresses were retrieved from the ASTRID database, which has been collecting individual-level data on the Swedish population for over 50 years [23]. Using the LuTrans transport model [24] each individual within ASTRID was allocated to different

For peer review only - http://bmjopen.bmj.com/site/about/guidelines.xhtml

modes of commuting based on travel survey and registry data on car ownership. Regression functions were used to model the probability for each mode of travel between small statistical areas of Stockholm County. A total of 111, 487 individuals from the Stockholm County population register were identified as potential bicycle commuters based on geographic distance from home-to-work and expected bicycle speed based on age and sex. Eligibility required an estimated bicycle commute of 30 minutes or less each way [22 25].

Estimating health outcomes

Nilsson Sommar et al [26] estimated changes in morbidity and mortality associated with the above chronic diseases and traffic accidents under the scenario, for potential bicycle commuters, current bicycle commuters, and the general population. Firstly, estimated health impacts due to increased physical activity among potential bicycle commuters were quantified in terms of decreased risk of type 2 diabetes, myocardial infarction (MI), stroke, dementia, colon cancer, breast cancer and heart failure. Secondly, change in air pollution exposure is assumed to benefit current bicycle commuters and the general population and disadvantage additional bicycle commuters in terms of risk for asthma, lung cancer, type 2 diabetes, stroke and MI [26]. Thirdly, health effects of injuries attributable to traffic accidents under this scenario of 111,487 potential bicycle commuters are also estimated [27]. Estimated Disability Adjusted Life Years (DALYs) were calculated for each area of health impact based on the disability weights reported by WHO Global Burden of Disease 2004 updates and expected changes in disease incidence. The duration of the disease was calculated as the expected life length after the first disease incidence. Life table calculations using mortality data for Stockholm County were used to calculate expected remaining life years based on age and gender [26].

For peer review only - http://bmjopen.bmj.com/site/about/guidelines.xhtml

Estimating cost-effectiveness

Information on the budget allocation for the bicycle pathways was obtained from the Stockholm Traffic Planning Office [22] [28]. The expected life cycle for new bicycle paths constructed in 2018 is fifty years [29].

The investment period of 2018-2030 was used as a build-up time to reach the estimated amount of additional bicyclists. We estimate that yearly investment costs will vary due to political and other circumstances. The number of additional bicyclists estimated to start active commuting in a given year is a proportion, of the estimated total sample, based on that years investment allocation (approximately 15 million euros/per year). Health impacts were simultaneously estimated consistent with the proportion of additional bicyclists in a given year. After 2030 an estimated 111,487 additional bicyclists were expected to commute. The number of DALYs attributable to this transport mode change was modelled for the remaining years. Long term health care costs (added and averted) are estimated in accordance with the change in health outcomes attributed to: 1) increased physical activity by potential bicycle commuters; 2) air pollution change for potential bicycle commuters; 3) air pollution change for current bicycle commuters; 4) air pollution change for the general population, and 5) traffic accidents incurred by potential bicycle commuters. These costs are given both in absolute terms and as the proportion of the 2017 Stockholm Municipality health budget [30].

Health outcomes are calculated for the fifty year period 2018-2068 taking the time interval between exposure and realised health benefits into account. It is estimated for physical activity exposure that it takes two years for 50% of the health benefits and six years for 100% of health benefits to be realised for both MI and stroke [31], seventeen and twenty years respectively for 50% and 100% of the health benefits to be realised for dementia, breast and colon cancer [31] and 3.2 and 8 years respectively for 50% and 100% of the health benefits to be

For peer review only - http://bmjopen.bmj.com/site/about/guidelines.xhtml

realised for type 2 diabetes [32]. The impacts of air pollution and traffic injuries were both assumed to occur during the current year. The mode shift from car to bicycle was assumed to be the only change in exposure.

This cost-effectiveness analysis adopts the CHEERS guidelines for health economic modelling [33]. Costs are reported in euros (2017) and discounted at an annual rate of 3%. Figure 1 shows the steps undertaken.

Sensitivity analyses

Sensitivity analysis is undertaken for the same investment costs assuming that numbers of potential bicycle commuters are as few as 25% of the estimated sample. In addition, the sensitivity to a reduced health impact and health care cost is provided. Results were reported in terms of its effect on the cost-effectiveness ratio (CER).

Estimating long term health care costs

The literature was used to inform estimates of long term health care costs for type 2 diabetes, MI, stroke, heart failure, dementia, colon cancer, breast cancer, lung cancer and asthma. (Table 1.) For comparability with Sweden, only papers reporting studies in high-income countries in which health care costs were met by public funds, were sought. Inclusion required that study estimates were based on administrative data recording health care costs matched to clinical diagnoses. The search was conducted in *Google Scholar*, *PubMed* and *Web of Science* between 1995 and 2018. Search terms included the health condition of interest and *lifetime/long term costs.* As no studies were found that specifically reported lifetime health care costs for asthma, MI or cancer outcomes, these estimates are based on one-year average health care costs for asthma, six-year average for MI and five-year disease costs for cancer.

For peer review only - http://bmjopen.bmj.com/site/about/guidelines.xhtml

For peer review only

Estimates of the lifetime costs for bicycle related injuries were derived from a study conducted in the Netherlands which used national register and hospital data covering the period 1998 and 2012 [34]. Costs extracted from the literature are inflated by the consumer price index and reported in euros (€) at the 2017 base year.

For peer review only

For peer review only - http://bmjopen.bmj.com/site/about/guidelines.xhtml

Table 1. Chronic disease treatment costs per case derived from the literature used to calculate health care costs

Disease	Sample size	Sample population	Data collection	Perspective	Method	Costs per case*[1]	Disease duration	Reference
Type 2 Diabetes	1000	Newly diagnosed type 2 diabetes	2009-2010	Health care	Markov modelling	83 155	Life-time	Zhou et al.
Stroke	9064	All first stoke cases registered in the national register	2009	Health and societal	Aggregated costs	74 125	Life-time	Ghatnekar et. al
Dementia	111 811	Medicare beneficiaries ≥65years	1997-2005	Health care	Cohort-based simulations	13 765	Life-time	Yang et.al.
Myocardial infarction	41 210	Acute myocardial infarction incidences in Alberta, Canada	2004-2014	Health care	Cohort based aggregated costs	147 262	6-years	Tran et al.

[1] * Costs per case expressed in € 2017 value

For peer review only

For peer review only - http://bmjopen.bmj.com/site/about/guidelines.xhtml

Heart failure	1054	Olmstead county residents who fulfilled the special criteria's established for heart failure.	2006	Health care	Tiangand-Huang method	113 975	Life-time	Dunlay et.al, (30)
Breast cancer	77009	Cohort of elderly Medicare, newly diagnosed cancer patients	1998-2004	Health care	5-year aggregated costs	21 177	5-years	Yabroff et.al (31)
Colon cancer	44838	Cohort of elderly Medicare, newly diagnosed cancer patients	1998-2004	Health care	5-year aggregated costs	42, 446	5- years	Yabroff et.al, (31)
Lung cancer	54665	Cohort of elderly Medicare, newly diagnosed cancer patients	1998-2004	Health care	5-year aggregated costs	43, 211	5-years	Yabroff et.al, (31)
Asthma	49 668	Patients with ICD-10-GM asthma code	2010	Health care	Aggregated costs	49 528 [2]	1-year	Jacob et.al,(32)

[2] Multiplied by the average length lived with the disease

For peer review only - http://bmjopen.bmj.com/site/about/guidelines.xhtml

For peer review only

Control group: 248 340	extracted from German sickness fund.

For peer review only

For peer review only - http://bmjopen.bmj.com/site/about/guidelines.xhtml

Results

Investment cost

The net infrastructure cost, excluding communication and maintenance costs, is €101 million. Assuming a fifty year life cycle for the bicycle path, a €2 million annual investment gives an average annual cost of €908 per converter.

Changes in health care costs

The fall in chronic disease cases attributable to increased physical activity among additional bicycle commuters is estimated to save €562 million, or €241 million after discounting (3%) giving an average annual return of €11.2 million. (Figure 2).

Traffic generated air pollutants were measured in nitrogen oxide concentrations [35]**.** Lower health care costs attributed to decreased air pollution exposure among current bicycle commuters and the general population are estimated to save €1 million annually. This is offset by increased health care costs of €0.28 million for additional bicycle commuters who are assumed to experience increased exposure to air pollution. (Figure 2).

The annual additional health care costs for injuries sustained as a result of the increased accident risk among additional bicycle commuters is estimated at €1.76 million.

Stockholm Council could avert 5.1 % of its planned 2017 health care expenditure, or 3.7% after discounting [30].

Cost-effectiveness ratio

A CER is evaluated for the given scenario compared with no assumed change in bicycle commuting. The CER is calculated by subtracting investment costs from total estimated health

For peer review only - http://bmjopen.bmj.com/site/about/guidelines.xhtml

care savings and dividing by the estimated reduction of DALYs giving a CER of €9933/DALY averted (Table 2).

Table 2. Estimated change in health care costs and health impact for each area of impact

Exposure	Estimated change in health care costs (million €)	Estimated DALY change
Physical activity	562.6	35 060
Air pollution change among the general population	54.5	21 975
Air pollution change among additional bicyclist	-14.0	-7286
Air pollution change among current bicyclists	2.4	130
Traffic injuries	- 88.2	-8209
Sum	517.3	41 670

Sensitivity analysis

The scenario assumed 111,487 additional bicycle commuters. The sensitivity of the estimates was assessed by assuming fewer potential bicycle commuters. If only 25% of the 111, 487 potential bicycle commuters were to change their mode of transport, the CER would still result in a surplus of €2643/DALY averted (Figure 3). Even under the assumptions of a reduced health impact and lower expected reductions in health care costs, the investment remains highly cost-effective.

Discussion

This is the first health economic evaluation of this type to use an evidence-based scenario that includes information on geolocation, demography and commuting modes by origin and destination zone drawn from a population register. Investment costs were compared with estimated long term health care costs and savings attributed to increased physical activity,

For peer review only - http://bmjopen.bmj.com/site/about/guidelines.xhtml

For peer review only

change in air pollution exposure and accident risk. To the best of our knowledge this is also the first economic evaluation to include morbidity health impacts in all three areas of impact. The investment is cost-effective from a health care sector perspective. Estimated net savings are 5.1% of the Stockholm County current health care budget, or 3.7% after discounting.

Physical activity

The largest reduction in health care costs is attributed to increased physical activity. Previous health economic assessments using hypothetical scenarios in Europe have also demonstrated cost savings in the health care sector attributed to physical activity [16 17]. In the United States (US) Grabow et al. [36] showed that, in a population of 2 million across eleven metropolitan areas, if one-half of the commutes, of less than eight kilometer round trip, were made by bicycle instead of car, mortality would be reduced by 700 premature deaths. Based on the United States (US) statistical value of life this is equivalent to $US3.8 billion (2010 values) annually or $1900 per individual [36]. In our study the estimated cost saving attributed to reduced morbidity from increased physical activity is €562 million or €100 per annum per additional bicycle commuter.

The benefits from different types of physical acitivity are variable and not necessarily linear. In their systematic review and meta-analysis of 22 cohort studies including over 977,000 people, Woodcock et al [37] concluded that, compared with no activity, two and a half hours per week of moderate intensity activity was associated with a 19% reduction in mortality risk and seven hours per week of moderate activity reduced mortality risk by 24%. reported in a meta-analysis that the relative risk (RR) would be reduced by 19% if currently inactive person took up vigourous physical activity (2.5hours per week). In this study physical activity effects are

For peer review only - http://bmjopen.bmj.com/site/about/guidelines.xhtml

reported only for bicycling although adjustments were made for other likely types of physical activity based on individuals' assumed physical capacity adjusted for age and sex.

Air pollution

Some other health economic analyses have assessed the economic value of a reduction in morbidity and mortality due to a reduction in air pollution exposure. Lindsay et al [38] for example, estimated the effect of reduced air pollution exposure on mortality, numbers of active days, and acute cardiac and respiratory hospital admissions, by calculating cost per health event and the statistical value of life based on New Zealand willingness to pay studies. Grabow et al. [36] used a BenMAP cost-benefit regulatory analysis to address the potential benefits attributable to an air pollution reduction in the general population as a result of a transport mode change from car to bicycle for trips of eight kilometers or less in various US cities. Based on acute hospital costs attributable to reduced automobile exhaust and increased ozone exposure net benefits were estimated at $US 4.94 billion (2010 values).

To the best of our knowledge this economic evaluation is also the first to model the impact of a change in air pollution exposure within the general population as well as among current bicycle commuters. Reduced health care costs within the general population (€54 million) are offset by additional costs of €14million among additional bicycle commuters, giving a net freed opportunity cost of €40 million within the health care budget over the 50 year life length of the investment.

Injuries

In their health economic assessment of cycling promotion in Florence, Italy, Taddei et al [17] predicted decreased traffic accidents. Woodcock et al. [39] modelled the health impacts of active transport visions in England and Wales and also predicted a fall in road traffic and accidents

For peer review only - http://bmjopen.bmj.com/site/about/guidelines.xhtml

under scenarios of increased walking and cycling. However using the same general scenario for increased bicycling in Stockholm County, Nilsson et al. [27] estimated health loss through increased DALYs due to increased numbers and types of traffic accidents being the first major study to highlight the negative impacts of increased injuries and fatalities resulting from the shift from car to bicycle. A strength of the Nilsson study, the results of which are incorported into our analysis of the health impacts and costs of traffic injuries, is the use of nationally representative hospítal and police register data on injuries occured.

Head and spinal injuries costs were included in an analysis of the effects of increased active travel in urban England and Wales on the National Health Service.[16] However these estimates were four times higher than the estimates used here. The current work is based on a study by Scholten et al [34] which assumed traumatic brain injuries occurring in about 9% of bicycle related traffic accidents.

It has been claimed that 60% of the bicycle accidents occur due to poor infrastructure for bicycling, in Sweden [40] and one of the aims of the Stockholm Bicycle Plan was to increase bicycle safety by building infrastructure for active commuters [28]. Improved road infrastructure has been shown to reduce bicycle fatalities by 45% [41] and it has been suggested that increasing the number of bicyclists can also have a “safety in numbers” effect which reduces accident risk [42]. An OECD study [43] showed that Sweden had the world’s safest roads with the lowest number of fatal accidents per billion-cycled kilometers compared with other European countries.

For peer review only - http://bmjopen.bmj.com/site/about/guidelines.xhtml

Total economic benefits

Health care costs

This study shows a net benefit corresponding to 5.1% of the 2017 Stockholm County health care budget. Grabow et al. [36] reported a 5% reduction in health care cost attributable to a change in air pollution exposure and increased physical activity. In a scenario of daily biking of 3.4 kilometers among urban residents in England and Wales, Jarrett et al. [16] reported that the costs for England National Health Service would be reduced by 0.8% as a consequence of increased physical activity.

Economic assessment

It is assumed that investment in physical infrastructure would lead to increased physical activity levels, and flow-on cost reductions within the health care sector. These assumptions have been adopted by others undertaking similar economic evaluations in which investment in infrastructure to promote active transport is assumed to increase physical activity and reduce air pollution. For example, a study conducted in Dane County in the US reported a cost benefit ratio of 1.87 over a ten-year cycle attributed to a $US450 million investment in sidewalks [44]. Some researchers suggest that the saved opportunity costs could finance the investment. For example a study of air quality and exercise-related health benefits resulting from reduced car travel in the Midwestern US Grabow et al.[36] concluded that the estimated reduction in health care costs could finance the infrastructure change within one to ten years. In Italy Taddei et al. [17] estimated that health care costs saved due to a reduction in NCDs could cover 50 kilometers of new bicycle roads within seven to ten years in Florence.

For peer review only - http://bmjopen.bmj.com/site/about/guidelines.xhtml

Strengths and limitations

Strengths

Many previous health economic analyses of active transport interventions have used hypothetical or proposed active travel interventions, however the scenario this study is based on was constructed linking several register data that included home and work address coordinates [25]. Gender and age-specific expected bicycle speeds were used to assess individuals' capacity to commute by bicycle between home and work in 30 minutes or less [22] [25]. In their systematic review of active transport interventions that include physical activity benefits, Brown et al [45] reported a limited inclusion of health effects with few studies reporting reductions in morbidity as well as mortality. Morbidity reduction is captured in the DALY weights in this study and the impacts of mortality under this scenario have been estimated in a companion paper [26]. Additionally the sensitivity analysis showed that the intervention is highly cost-effective even if only one quarter of the potential bicycle commuters were to take up bicycle commuting.

Limitations

The health impact estimates our economic assessment are based on the Global Burden of Disease [46]. We have not included all possible health impacts. We did not, for example, account for impacts on mental health (e.g. depression) and obesity that are expected to result from increased physical activity [47 48]. A study in the US reported that obesity reductions resulting from active transport could save $90.93 million in health care costs [12].

It is possible that health benefits and cost savings are underestimated in this study due to our not fully accounting for the benefits of reduced obesity and improved well-being that may accrue over time and flow-on to the broader community. These effects would be considered

For peer review only - http://bmjopen.bmj.com/site/about/guidelines.xhtml

For peer review only

in an economic evaluation conducted from a societal perspective, which is beyond the scope of the present analysis. There are other potential savings which were not included. For example, it has been shown that people value the time bicycling to work more than the time spent in motorized transport [49]. If a societal perspective had been applied cost-effectiveness may have been higher.

Except for the effect of the mode-shift towards increased bicycling, no other changes in air pollution concentrations were assumed. In terms of air pollution effects, the US Environmental Protection Agency applies segmented lag-times for mortality estimates [50]. However, due to the lack of studies on lag times for air pollution effects on morbidity, it was assumed that effects occurred in the same year as exposure.

In using secondary data to estimate long term costs for chronic NCDs there was a lack of evidence for cancer and asthma patients. Estimates are therefore based on best approximations from scientific evidence published in the peer-reviewed literature. Although it is common practice for economic evaluations to draw upon evidence from previous studies, comparing evidence from studies with different methodological approaches in different settings is not without its shortcomings [51]. Studies from other high-income countries were used so that the results could be reasonably generalised to Sweden.

Conclusions

From a health care perspective investing in urban infrastructure to increase bicycling in Stockholm is extremely cost-effective in relation to the Swedish threshold value of € 53,000 per DALY averted. This evaluation makes best use of available data and calculates estimates based on a previously developed realistic scenario. More work of this type is needed in order

For peer review only - http://bmjopen.bmj.com/site/about/guidelines.xhtml

to build an evidence base for decision-makers responsible for investing funds in public infrastructure.

Author Contributions

HKK planned the health economic study and undertook the data collection and economic analysis with input from LL. HKK wrote the first draft. JSW reviewed the overall content, provided input and revised and re-drafted the paper in conjunction with HKK. BF advised on the planning and conduct of the study and was responsible for the overall transport scenario project of which this work was part. JNS provided health impact estimates and critical input into the planning and conduct of the study, assisted with the statistical analyses, and helped revise the drafts. All authors read and approved the final version of the manuscript.

Role of the funding source

This work was funded by the Swedish Research Council for Health, Working Life and Welfare (grant number 2012-1296). The paper reflect only the authors views, and the funder had no role in the design or conduct of the study.

Competing interests

All authors have completed the ICMJE uniform disclosure form at www.icmje.org/coi_disclosure.pdf and declare: no support from any organization for the submitted work; no financial relationships with any organizations that might have an interest in the submitted work in the previous three years; no other relationships or activities that could appear to have influenced the submitted work.

For peer review only

For peer review only - http://bmjopen.bmj.com/site/about/guidelines.xhtml

Transparency declaration

The lead author affirms that the manuscript is an honest, accurate, and transparent account of the study being reported; that no important aspects of the study have been omitted; and that any discrepancies from the study as planned have been explained.

Patient and Public involvement: No patient or public involvement.

The datasets analyzed during the current study are available from the corresponding author on reasonable request.

Acknowledgements

Bertil Forsberg received a grant (2012-1296) from FORTE (Forskningsrådet för hälsa, arbetsliv och välfärd) for the transport scenario study. We thank study leaders and co-authors in the transport scenario study, in particular Peter Schantz, Christer Johansson, Wasif Raza and Magnus Strömgren.

Figure legends

Figure 1. Flowchart for estimated chain of events as the base for conducting the cost-effectiveness analysis

Figure 2. Estimated yearly expenditure averted in the health care sector due to increased physical activity, change in air pollution concentrations and risk of traffic injuries

Figure 3. Sensitivity analysis of the CER considering number of obtained bicyclists, the health impact, and health care costs

Data sharing statement: No additional data available.

For peer review only - http://bmjopen.bmj.com/site/about/guidelines.xhtml

For peer review only

References

1. World Health Organization. 2008-2013 Action Plan for the Global Strategy for the Prevention and Control of Noncommunicable Diseases. Genève, 2008:48.

2. Cecchini M, Sassi F, Laver JA, et al. Tackling of unhealthy diets, physical inactivity, and obesity: health effects and cost-effectiveness. *The Lancet* 2010;376:1775-84. doi: DOI:10.1016/S0140-6736(10)61514-0

3. World Health Organization. Global recommendations on physical activity for health. Genève, 2010:60.

4. Lee I-M, Shiroma EJ, Lobelo F, et al. Effect of physical inactivity on major non-communicable diseases worldwide: an analysis of burden of disease and life expectancy. *The Lancet* 2012;380:219-29. doi: http://dx.doi.org/10.1016/50140-6736(12)61031-9

5. World Health Organization, OECD, European Observatory. Promoting health, preventing disease. The economic case. In: Davis McDaid FS, Sherry Merkur, ed. New York, 2015:370.

6. World Health Organization. Physical Activity Fact Sheet. 2016

7. Cecchini M, Bull F. Promoting physical activity. In: David McDaid FS, Sherry Merkur, ed. Promoting health, preventing disease The economic case. New York: McGraw Hill Education 2015:101-34.

8. World Health Organization. Urbanisation and Health. *Bulletin of the World Health Organization* 2010;88:245-46.

9. United Nations. Sustainable Development Goals. 2015

10. IPCC. Climate change 2007 - Mitigation of climate change. 2007

11. World Health Organization Regional Office for Europe, OECD. Economic cost of the health impact of air pollution in Europe: clean air, health and wealth. Copenhagen, 2015:66.

12. Pucher J, Buehler R, Bassett DR, et al. Walking and cycling to health: a comparative analysis of city, state, and international data. *Am J Public Health* 2010;100(10):1986-92. doi: 10.2105/ajph.2009.189324 [published Online First: 2010/08/21]

13. Fraser SD, Lock K. Cycling for transport and public health: a systematic review of the effect of the environment on cycling. *Eur J Public Health* 2011;21(6):738-43. doi: 10.1093/eurpub/ckq145 [published Online First: 2010/10/12]

14. Cobiac LJ, Vos T, Barendregt JJ. Cost-effectiveness of interventions to promote physical activity: a modelling study. *PLoS Med* 2009;6(7):e1000110. doi: 10.1371/journal.pmed.1000110 [published Online First: 2009/07/15]

15. Mulley C, Tyson R, McCue P, et al. Valuing active travel: including the health benefits of sustainable transport in transportation appraisal frameworks. *Research in Transportation Business and Management* 2013;7:27-34.

16. Jarrett J, Woodcock J, Griffiths UK, et al. Effect of increasing active travel in urban England and Wales on costs to the National Health Service. *The Lancet* 2012;379(9832):2198-205. doi: 10.1016/s0140-6736(12)60766-1 [published Online First: 2012/06/12]

17. Taddei C, Gnesotto R, Forni S, et al. Cycling promotion and non-communicable disease prevention: health impact assessment and economic evaluation of cycling to work or school in Florence. *PLoS One* 2015;10(4):e0125491. doi: 10.1371/journal.pone.0125491 [published Online First: 2015/05/01]

For peer review only - http://bmjopen.bmj.com/site/about/guidelines.xhtml

18. Beale SJ, Bending MW, Trueman P, et al. Should we invest in environmental interventions to encourage physical activity in England? An economic appraisal. *Eur J Public Health* 2012;22(6):869-73. doi: https://doi.org/10.1093/eurpub/ckr151

19. Hanson S, Jones A. Missed opportunities in the evaluation of public health interventions: a case study of physical activity programmes. *BMC Public Health* 2017;17(1):674. doi: 10.1186/s12889-017-4683-z [published Online First: 2017/08/24]

20. Weatherly H, Drummond M, Claxton K, et al. Methods for assessing the cost-effectiveness of public health interventions: key challenges and recommendations. *Health Policy* 2009;93(2-3):85-92. doi: 10.1016/j.healthpol.2009.07.012 [published Online First: 2009/08/28]

21. OECD/EU. Health at a Glance: Europe 2016 - State of Health in the EU Cycle. Paris, 2016:204.

22. Strömgren M, Schantz. P, Nilsson Sommar J, et al. Modelling a commuter modal shift from car trips to cycling: Scenario construction and outcomes for Stockholm County, Sweden. To be submitted., 2019.

23. Stjenström O. The Astrid data base and population geography (only in Swedish "Databasen ASTRID och befolkningsgeografi exemplen integration och barnfamiljenas geografi" *Geografiska Notised*;60. No.2

24. Jonsson D BS, Almström P, Algers S. The usefulness of transport models in Swedish planning practice. *Transport Reviews* 2011;31(2):251-65.

25. Nilsson Sommar J, Schantz P, Strömgren M, et al. The potential for reduced mortality by increased cycle commuting - a health impact assessment using register data on home and work addresses in Stockholm, Sweden. To be submitted., 2019.

26. Nilsson Sommar J, Johansson C, Lövenheim B, et al. Overall health impacts of a potential increase in active commuting in Stockholm, Sweden. To be submitted., 2019.

27. Nilsson P, Stigson H, Ohlin M, et al. Modelling the effect on injuries and fatalities when changing mode of transport from car to bicycle. *Accid Anal Prev* 2017;100:30-36. doi: 10.1016/j.aap.2016.12.020 [published Online First: 2017/01/14]

28. En el av Framkomlighetsstrategin: Cyckelplan. In: Stad S, ed., 2016.

29. Livslängs och Trafikanalys. In: Trafikverket, ed., 2002:6.

30. Stockholm Läns Lansting Hälsa- och sjukvårdsnämden. Slutlig budget 2017 HSN: Förändringsarbete i fokus när vården i länet växer, 2017.

31. Kahlmeier S CN DH, et al. . Health economic assessment tools (HEAT) for walking and cycling: methodology and user guide. *Copenhagen: WHO Regional Office Europe* 2011

32. Umpierre D, Ribeiro PA, Kramer CK, et al. Physical activity advice only or structured exercise training and association with HbA1c levels in type 2 diabetes: a systematic review and meta-analysis. *JAMA* 2011;305(17):1790-9. doi: 10.1001/jama.2011.576 [published Online First: 2011/05/05]

33. Husereau D, Drummond M, Petrou S, et al. Consolidated Health Economic Evaluation Reporting Standards (CHEERS)--explanation and elaboration: a report of the ISPOR Health Economic Evaluation Publication Guidelines Good Reporting Practices Task Force. *Value Health* 2013;16(2):231-50. doi: 10.1016/j.jval.2013.02.002 [published Online First: 2013/03/30]

34. Scholten AC, Polinder S, Panneman MJ, et al. Incidence and costs of bicycle-related traumatic brain injuries in the Netherlands. *Accid Anal Prev* 2015;81:51-60. doi: 10.1016/j.aap.2015.04.022 [published Online First: 2015/05/06]

For peer review only

35. Johansson C, Lovenheim B, Schantz P, et al. Impacts on air pollution and health by changing commuting from car to bicycle. *Sci Total Environ* 2017;584-585:55-63. doi: 10.1016/j.scitotenv.2017.01.145 [published Online First: 2017/01/31]

36. Grabow ML, Spak SN, Holloway T, et al. Air quality and exercise-related health benefits from reduced car travel in the midwestern United States. *Environ Health Perspect* 2012;120(1):68-76. doi: 10.1289/ehp.1103440 [published Online First: 2011/11/04]

37. Woodcock J, Franco OH, Orsini N, et al. Non-vigorous physical activity and all-cause mortality: systematic review and meta-analysis of cohort studies. *Int J Epidemiology* 2011;40(1):121-38. doi: 10.1093/ije/dyq104 [published Online First: 2010/07/16]

38. Lindsay G, Macmillan A, Woodward A. Moving urban trips from cars to bicycles: impact on health and emissions. *Australian and New Zealand Journal of Public Health* 2011;35(1):54-60. doi: 10.1111/j.1753-6405.2010.00621.x [published Online First: 2011/02/09]

39. Woodcock J, Givoni M, Morgan AS. Health impact modelling of active travel visions for England and Wales using an Integrated Transport and Health Impact Modelling Tool (ITHIM). *PLoS One* 2013;8(1):e51462. doi: 10.1371/journal.pone.0051462 [published Online First: 2013/01/18]

40. Niska A G, S, Nyberg, J, Eriksson, J. . Cyklisters singelolyckor - Analys av olycks- och skadedata samt djupintervjuer (sIngle bicycle accidents analysis of hospital injury data and interviews). *VTI, Linköping, Sweden* 2013

41. Schepers PM, Rob; Wegman, Fred; Heinen, Eva, . Road safety and bicycle usage impacts of unbundling vehicular and cycle traffic in Dutch urban networks. *European Journal of Transport and Infrastructure Research* 2013;13(3):221-38.

42. Jacobsen PL. Safety in numbers: more walkers and bicyclists, safer walking and bicycling. *Inj Prev* 2015;21(4):271-5. doi: 10.1136/ip.9.3.205rep [published Online First: 2015/07/24]

43. OECD/EU. Road Safety Annual Report 2011. OECD International Transport Forum, Paris, 2012.

44. Guo JY, Gandavarapu S. An economic evaluation of health-promotive built environment changes. *Prev Med* 2010;50 Suppl 1:S44-9. doi: 10.1016/j.ypmed.2009.08.019 [published Online First: 2009/10/21]

45. Brown V, Diomedi, B., Moodie, M., Veerman, J., & Carter, R. A systematic review of economic analyses of active transport interventions that include physical activity benefits. *Transport Policy* 2016;45(C):190-208. doi: 10.1016/j.tranpol.2015.10.003

46. World Health Organization. Global burden of disease 2004 update: disability weights for diseases and conditions. Genève: WHO, 2004:9.

47. Gill JM, Malkova D. Physical activity, fitness and cardiovascular disease risk in adults: interactions with insulin resistance and obesity. *Clinical Science (London, England : 1979)* 2006;110(4):409-25. doi: 10.1042/cs20050207 [published Online First: 2006/03/11]

48. Warburton DE, Nicol CW, Bredin SS. Health benefits of physical activity: the evidence. *CMAJ : Canadian Medical Association Journal = journal de l'Association medicale canadienne* 2006;174(6):801-9. doi: 10.1503/cmaj.051351 [published Online First: 2006/03/15]

49. Borjesson M EJ. The value of time and external benefits in bicycle appraisal. *Transport Research Part A Policy and Practice* 2012;46(4):673-83.

50. U.S. Environmental Protection Agency Office of Air Quality Planning and Standards Health and Environmental Impacts Division. Regulatory Impact Analysis for the Final Revisions to the National Ambient Air Quality Standards for Particulate Matter. EPA-452/R-12-005. Research Triangle Park, NC 27711, 2012:474.

For peer review only - http://bmjopen.bmj.com/site/about/guidelines.xhtml

51. Briggs A CC, Sculpher M. Decision Modelling for Health Economic Evaluation. Oxford University Press, 2011.

For peer review only

For peer review only - http://bmjopen.bmj.com/site/about/guidelines.xhtml

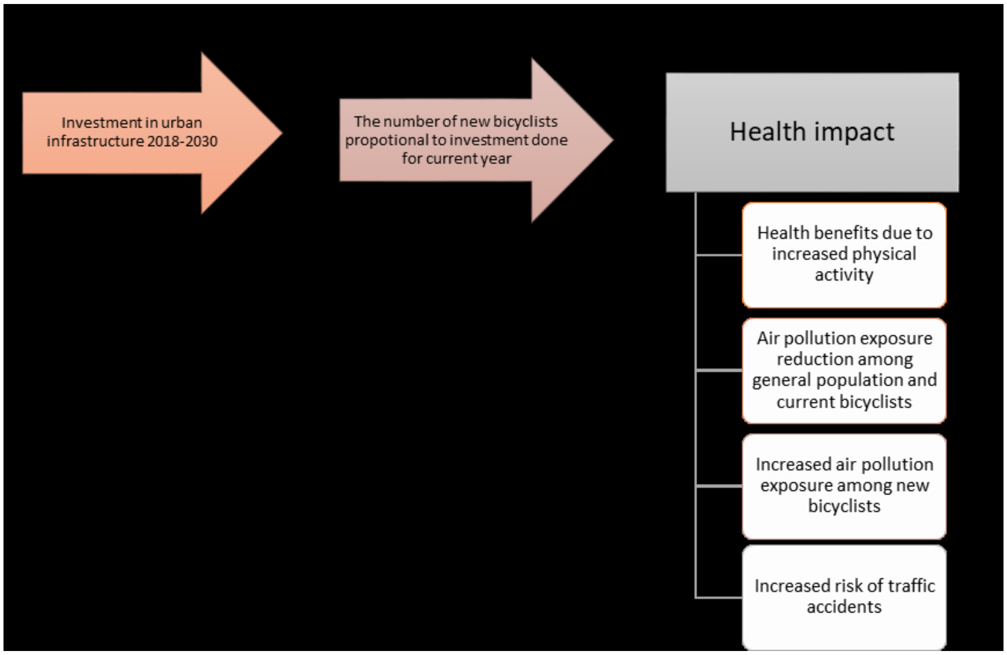

Figure 1. Flowchart for estimated chain of events as the base for conducting the cost-effectivness analysis

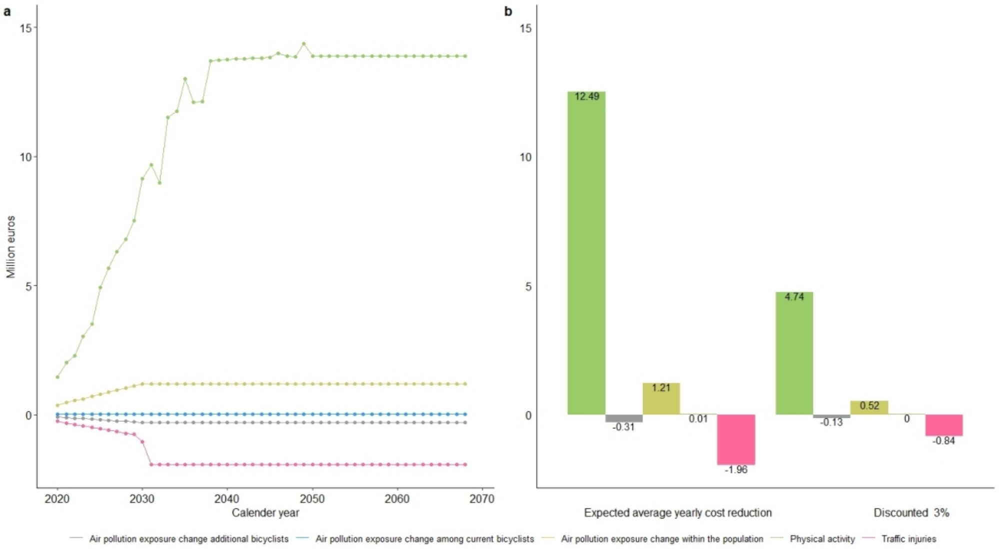

Figure 2. Estimated yearly expenditure averted in the health care sector due to increased physical activity, change in air pollution concentrations and risk of traffic injuries

383x211mm (96 x 96 DPI)

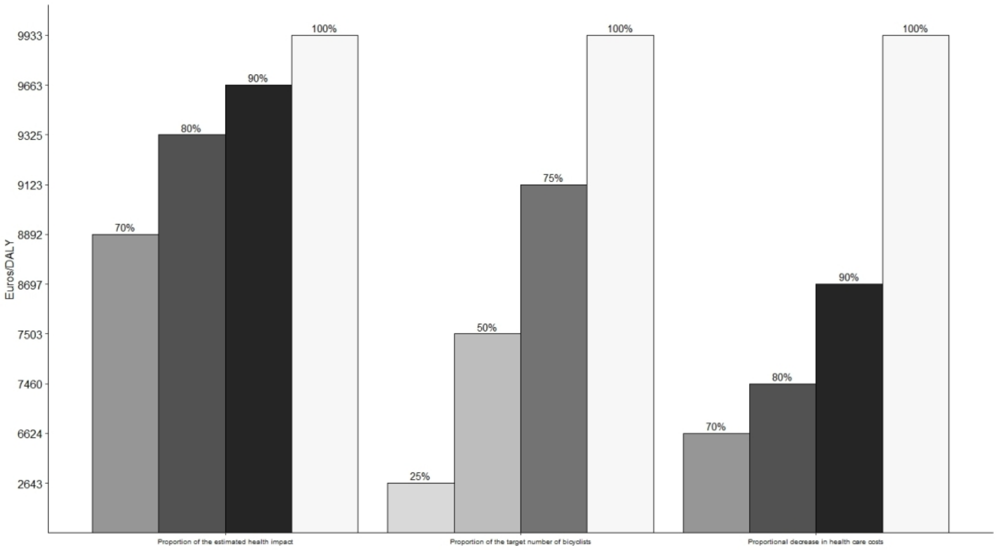

Figure 3. Sensitivity analysis of the CER considering number of obtained bicyclists, the health impact, and health care costs

383x211mm (96 x 96 DPI)

CHEERS Checklist
Items to include when reporting economic evaluations of health interventions

The **ISPOR CHEERS Task Force Report**, *Consolidated Health Economic Evaluation Reporting Standards (CHEERS)—Explanation and Elaboration: A Report of the ISPOR Health Economic Evaluations Publication Guidelines Good Reporting Practices Task Force*, provides examples and further discussion of the 24-item CHEERS Checklist and the CHEERS Statement. It may be accessed via the *Value in Health* or via the ISPOR Health Economic Evaluation Publication Guidelines – CHEERS: Good Reporting Practices webpage: http://www.ispor.org/TaskForces/EconomicPubGuidelines.asp

For peer review only

Section/item	Item No	Recommendation	Reported on page No/ line No
**Title and abstract**			
Title	1	Identify the study as an economic evaluation or use more specific terms such as "cost-effectiveness analysis", and describe the interventions compared.	1/1-2
Abstract	2	Provide a structured summary of objectives, perspective, setting, methods (including study design and inputs), results (including base case and uncertainty analyses), and conclusions.	2-3/25
**Introduction**			
Background and objectives	3	Provide an explicit statement of the broader context for the study. Present the study question and its relevance for health policy or practice decisions.	5/108-111
**Methods**			
Target population and subgroups	4	Describe characteristics of the base case population and subgroups analysed, including why they were chosen.	6/127
Setting and location	5	State relevant aspects of the system(s) in which the decision(s) need(s) to be made.	NA
Study perspective	6	Describe the perspective of the study and relate this to the costs being evaluated.	6/116
Comparators	7	Describe the interventions or strategies being compared and state why they were chosen.	NA
Time horizon	8	State the time horizon(s) over which costs and consequences are being evaluated and say why appropriate.	6/122
Discount rate	9	Report the choice of discount rate(s) used for costs and outcomes and say why appropriate.	9/183
Choice of health outcomes	10	Describe what outcomes were used as the measure(s) of benefit in the evaluation and their relevance for the type of analysis performed.	7/150
Measurement of effectiveness	11a	*Single study-based estimates:* Describe fully the design features of the single effectiveness study and why the single study was a sufficient source of clinical effectiveness data.	NA

	11b	*Synthesis-based estimates:* Describe fully the methods used for identification of included studies and synthesis of clinical effectiveness data.	9/186
Measurement and valuation of preference based outcomes	12	If applicable, describe the population and methods used to elicit preferences for outcomes.	NA
Estimating resources and costs	13a	*Single study-based economic evaluation:* Describe approaches used to estimate resource use associated with the alternative interventions. Describe primary or secondary research methods for valuing each resource item in terms of its unit cost. Describe any adjustments made to approximate to opportunity costs.	NA
	13b	*Model-based economic evaluation:* Describe approaches and data sources used to estimate resource use associated with model health states. Describe primary or secondary research methods for valuing each resource item in terms of its unit cost. Describe any adjustments made to approximate to opportunity costs.	10/203
Currency, price date, and conversion	14	Report the dates of the estimated resource quantities and unit costs. Describe methods for adjusting estimated unit costs to the year of reported costs if necessary. Describe methods for converting costs into a common currency base and the exchange rate.	8/156 Figure 1
Choice of model	15	Describe and give reasons for the specific type of decision-analytical model used. Providing a figure to show model structure is strongly recommended.	8/156
Assumptions	16	Describe all structural or other assumptions underpinning the decision-analytical model.	8/173
Analytical methods	17	Describe all analytical methods supporting the evaluation. This could include methods for dealing with skewed, missing, or censored data; extrapolation methods; methods for pooling data; approaches to validate or make adjustments (such as half cycle corrections) to a model; and methods for handling population heterogeneity and uncertainty.	9/182
**Results**			
Study parameters	18	Report the values, ranges, references, and, if used, probability distributions for all parameters. Report reasons or sources for distributions used to represent uncertainty where appropriate. Providing a table to show the input values is strongly recommended.	11-13/Tabel 1.
Incremental costs and outcomes	19	For each intervention, report mean values for the main categories of estimated costs and outcomes of interest, as well as mean differences between the comparator groups. If applicable, report incremental cost-effectiveness ratios.	14/231 Tabel 2
Characterising uncertainty	20a	*Single study-based economic evaluation:* Describe the effects of sampling uncertainty for the estimated incremental cost and incremental effectiveness parameters, together with the impact	NA

For peer review only

		of methodological assumptions (such as discount rate, study perspective).	
	20b	*Model-based economic evaluation:* Describe the effects on the results of uncertainty for all input parameters, and uncertainty related to the structure of the model and assumptions.	9/186
Characterising heterogeneity	21	If applicable, report differences in costs, outcomes, or cost-effectiveness that can be explained by variations between subgroups of patients with different baseline characteristics or other observed variability in effects that are not reducible by more information.	NA
**Discussion**			
Study findings, limitations, generalisability, and current knowledge	22	Summarise key study findings and describe how they support the conclusions reached. Discuss limitations and the generalisability of the findings and how the findings fit with current knowledge.	15/241
**Other**			
Source of funding	23	Describe how the study was funded and the role of the funder in the identification, design, conduct, and reporting of the analysis. Describe other non-monetary sources of support.	22/386-390
Conflicts of interest	24	Describe any potential for conflict of interest of study contributors in accordance with journal policy. In the absence of a journal policy, we recommend authors comply with International Committee of Medical Journal Editors recommendations.	

For consistency, the CHEERS Statement checklist format is based on the format of the CONSORT statement checklist

The **ISPOR CHEERS Task Force Report** provides examples and further discussion of the 24-item CHEERS Checklist and the CHEERS Statement. It may be accessed via the *Value in Health* link or via the ISPOR Health Economic Evaluation Publication Guidelines – CHEERS: Good Reporting Practices webpage: http://www.ispor.org/TaskForces/EconomicPubGuidelines.asp

The citation for the CHEERS Task Force Report is:
Husereau D, Drummond M, Petrou S, et al. Consolidated health economic evaluation reporting standards (CHEERS)—Explanation and elaboration: A report of the ISPOR health economic evaluations publication guidelines good reporting practices task force. Value Health 2013;16:231-50.

For peer review only

BMJ Open

Health economic assessment of a scenario to promote bicycling as active transport in Stockholm, Sweden

Journal:	*BMJ Open*
Manuscript ID	bmjopen-2019-030466.R2
Article Type:	Research
Date Submitted by the Author:	06-Aug-2019
Complete List of Authors:	Kriit, Hedi; Umeå Universitet Medicinska fakulteten, Sustainable Health<br>Williams, Jennifer Stewart; Umeå Universitet Medicinska fakulteten, Department of Epidemiology and Global Health<br>Lindholm, Lars; Umeå Universitet Medicinska fakulteten, Department of Epidemiology and Global Health<br>Forsberg, Bertil; Umeå University, Public Health and Clinical Medicine<br>Sommar, Johan; Umea University Department of Public Health and Clinical Medicine, Sustainable health
**Primary Subject Heading**:	Health economics
Secondary Subject Heading:	Health economics, Health policy, Public health
Keywords:	HEALTH ECONOMICS, DALY, active commuting, Health impacts, Cost-effectiveness analysis, Transport mode shift

SCHOLARONE™
Manuscripts

Health economic assessment of a scenario to promote bicycling as active transport in Stockholm, Sweden

Hedi Katre Kriit[1] Jennifer Stewart Williams[2 3] Lars Lindholm[2] Bertil Forsberg[1] Johan Nilsson Sommar[1]

[1] Section of Sustainable Health
Department of Public Health and Clinical Medicine
Umeå University
SE-90185, Umeå
Sweden

[2] Department of Epidemiology and Global Health
Umeå University
SE-90187, Umeå
Sweden

[3] Research Centre for Generational Health and Ageing
Faculty of Health, University of Newcastle
New Lambton Heights, NSW 2305
Australia

Corresponding and first author:
Hedi Katre Kriit
Email: Hedi.Kriit@umu.se

Key words: **health economic evaluation, commuting, transport mode shift, health impacts, DALY**

Word count: **4038**

iew only

For peer review only - http://bmjopen.bmj.com/site/about/guidelines.xhtml

Abstract

Objectives

To conduct a health economic evaluation of a proposed investment in urban bicycle infrastructure in Stockholm County, Sweden.

Design

A cost-effectiveness analysis is undertaken from a health care perspective. Investment costs over a 50-year life cycle are offset by averted health care costs and compared with estimated long term impacts on morbidity, quantified in Disability Adjusted Life Years (DALYs). The results are re-calculated under different assumptions to model the effects of uncertainty.

Setting

The Municipality of Stockholm (population 2.27 million) committed funds for bicycle path infrastructure with the aim of achieving a 15% increase in the number of bicycle commuters by 2030. This work is based on a previously constructed scenario, in which individual registry data on home and work address and a transport model allocation to different modes of transport identified 111,000 individuals with the physical capacity to bicycle to work within 30 minutes but that currently drive a car to work.

Results

Morbidity impacts and health care costs attributed to increased physical activity, change in air pollution exposure and accident risk are quantified under the scenario. The largest reduction in health care costs is attributed to increased physical activity and the second largest to reduced air pollution exposure among the population of Greater Stockholm. The expected net benefit from the investment is 8.7% of the 2017 Stockholm County health care budget, and

For peer review only - http://bmjopen.bmj.com/site/about/guidelines.xhtml

For peer review only

3.7% after discounting. The economic evaluation estimates that the intervention is cost-effective and each DALY averted gives a surplus of €9933. The results remained robust under varied assumptions pertaining to reduced numbers of additional bicycle commuters.

Conclusion

Investing in urban infrastructure to increase bicycling as active transport is cost-effective from a health care sector perspective.

Article summary

Strengths and limitations

- This transport mode shift scenario is based on registry data and reflects potential outcomes from increased bicycling in Stockholm County.
- Due to data limitations, it was not possible to model possible changes in population health in the absence of the intervention.
- Investment costs are compared with the expected reduction of chronic disease related health care costs due to the transport mode shift.
- Secondary data were used to estimate the life-time costs of chronic diseases.

For peer review only

For peer review only - http://bmjopen.bmj.com/site/about/guidelines.xhtml

Background

Four chronic illnesses – cardiovascular disease, cancer, type 2 diabetes and respiratory disorders - account for at least 60% of global deaths. Several known modifiable lifestyle behaviours such as tobacco smoking, unhealthy diets, harmful alcohol use and physical inactivity impact on these chronic illness through associations with risk factors such as obesity, hypertension and high blood sugar levels [1-6]. Physical inactivity is the fourth most important risk factor for premature mortality in the European Union after tobacco smoking, high blood pressure and overweight [5].

There is now widespread evidence of the importance of physical activity in promoting and maintaining good health [3 5 6]. Even the relatively inactive can benefit from marginal increases in physical activity [7]. Since 2010, when the first official set of global recommendations for physical activity were launched by the World Health Organization (WHO)[3], policies to address insufficient physical activity have been implemented in over half WHO Member States [6].

Currently half of the world's population live in urban environments and this proportion is expected to reach 60% by 2030 [8 9]. Participation in physical activity can be difficult in cities where the heavy use of motorized transportation is estimated to be responsible for 70% of environmental urban air pollution and 13% of global green gas emissions [10]. In 2010 road transport contributed 40% of the economic costs of premature deaths from air pollution in the WHO European Region [11].

Active transport through, for example, bicycling as a way of commuting to work, has been suggested as a way of increasing physical activity and reducing motorised traffic emissions in urban areas. However the health gains for those switching from car to bike transport in heavy traffic areas are offset by the increased risk of serious injury and greater exposure to poor air

For peer review only - http://bmjopen.bmj.com/site/about/guidelines.xhtml

quality. Furthermore the distribution of costs and benefits is not equally spread across socio-economic and demographic groups [7 12 13].

In a study of the cost-effectiveness of interventions to encourage more physical activity in the Australian population, Cobiac et al reported that a package of physical activity interventions that include active transport could yield both public health benefits and health sector cost savings [14]. Another study in Australia estimated that health care costs would be reduced by $A1.12 per bicycled kilometer [15]. Studies using previously published disease specific health care costs have estimated significant cost reductions based on hypothetical scenarios of increased active commuting in the United Kingdom [16] and Italy [17]. For example, Jarret et al. estimated that the National Health Service could save roughly £17 billion within 20 years due to reduced prevalence of seven non-communicable diseases (NCDs) resulting from increased walking and bicycling [16]. A number of economic assessments have suggested that travel/transport-related interventions are cost-effective in terms of costs per healthy life-year gained in adult populations [7 18]. Although, not without methodological challenges, there is a need to evaluate the costs and benefits of interventions that promote physical activity through active transport from a health sector perspective [19 20].

In 2015 NCDs accounted for €115 billion in health care costs in European countries [21]. Strategies that address physical inactivity can deliver public health gains through the prevention of chronic diseases. Active transport strategies involving a shift from motor transport can also have wider benefits for the population through improvements in air quality. Sound economic evaluations are needed to build an evidence base in this area [2].

This paper presents a health economic evaluation of a proposed investment in urban bicycle infrastructure in Stockholm, Sweden, from a health care sector perspective. The long term

For peer review only - http://bmjopen.bmj.com/site/about/guidelines.xhtml

change in outcomes and health care costs are quantified within a scenario of increased commuting by bicycle and cost-effectiveness is derived in relation to the planned investment.

Methods

Evaluation scenario

As part of its 2018-2030 plan, the Municipality of Stockholm (population 2.27 million) committed funds for bicycle path infrastructure with the aim of achieving a 15% increase in bicycle commuters by 2030. Based on the previously developed transport shift scenario it is estimated that 111,487 current car drivers could potentially switch to bicycle commuting between home and work. Thereafter, in the economic evaluation it is assumed that yearly investment costs lead to a proportional amount of new bicyclists from the estimated number of potential converters. Health impacts, attributed to increased physical activity, differential exposure to air pollution and traffic accidents, and changes in health care costs are evaluated for potential bicycle commuters, current bicycle commuters and the general population. The timeline assumes that it takes 12 years of investment for all estimated potential bicyclists to shift their mode of transport and health impacts are calculated for a fifty year life length of the investment in bicycle path infrastructure [22]. Yearly health impacts are compared, ceteris paribus, with current 2017 register data on disease incidences that were assumed to be constant during the fifty years of assessment. The corresponding estimated lifetime health care costs were calculated and used as a comparator for cost effectiveness analysis.

Population base

The study population is based on a scenario constructed for a previous study in which data on geolocation, demography and commuting modes by origin and destination zone were referenced [22]. Home and workplace addresses were retrieved from the ASTRID database,

For peer review only - http://bmjopen.bmj.com/site/about/guidelines.xhtml

which has been collecting individual-level data on the Swedish population for over 50 years [23]. Using the LuTrans transport model [24] each individual within ASTRID was allocated to different modes of commuting based on travel survey information and registry data on car ownership. Regression functions were used to model the probability for each mode of travel between small statistical areas of Stockholm County. A total of 111,487 individuals from the Stockholm County population register who currently drive a car to work were identified as potential bicycle commuters based on geographic distance from home-to-work and expected bicycle speed based on age and sex. Eligibility required an estimated bicycle commute of 30 minutes or less each way [22 25].

Estimating health outcomes

Nilsson Sommar et al [26] estimated changes in morbidity and mortality associated with the above chronic diseases and traffic accidents under the scenario, for potential bicycle commuters, current bicycle commuters, and the general population. Firstly, estimated health impacts due to increased physical activity among potential bicycle commuters were quantified in terms of decreased risk of type 2 diabetes, myocardial infarction (MI), stroke, dementia, colon cancer, breast cancer and heart failure. Secondly, change in air pollution exposure is assumed to benefit current bicycle commuters and the general population and disadvantage additional bicycle commuters in terms of risk for asthma, lung cancer, type 2 diabetes, stroke and MI [26]. Thirdly, health effects of injuries attributable to traffic accidents under this scenario of 111,487 potential bicycle commuters are also estimated [27]. Estimated Disability Adjusted Life Years (DALYs) were calculated for each area of health impact based on the disability weights reported by WHO Global Burden of Disease 2004 updates and expected changes in disease incidence. The duration of the disease was calculated as the expected life length after

For peer review only - http://bmjopen.bmj.com/site/about/guidelines.xhtml

the first disease incidence. Life table calculations using mortality data for Stockholm County were used to calculate expected remaining life years based on age and gender [26].

Estimating cost-effectiveness

Information on the budget allocation for the bicycle pathways was obtained from the Stockholm Traffic Planning Office [22 28]. The expected life cycle for new bicycle paths constructed in 2018 is fifty years [29].

The investment period of 2018-2030 was used as a build-up time to reach the estimated amount of additional bicyclists. We estimate that yearly investment costs will vary due to political and other circumstances. The number of additional bicyclists estimated to start active commuting in a given year is a proportion of the estimated potential bicycle commuters, based on that year's investment allocation (approximately 15 million euros per year). Health impacts were simultaneously estimated consistent with the proportion of additional bicyclists in a given year. After 2030 an estimated 111,487 additional bicyclists were expected to commute. The number of DALYs attributable to this transport mode change was modelled for the remaining years. Long term health care costs (added and averted) are estimated in accordance with the change in health outcomes attributed to: 1) increased physical activity by potential bicycle commuters; 2) air pollution change for potential bicycle commuters; 3) air pollution change for current bicycle commuters; 4) air pollution change for the general population, and 5) traffic accidents incurred by potential bicycle commuters. These costs are given both in absolute terms and as the proportion of the 2017 Stockholm Municipality health budget [30].

Health outcomes are calculated for the fifty-year period 2018-2068 taking the time interval between exposure and realised health benefits into account. It is estimated for physical activity exposure that it takes two years for 50% of the health benefits and six years for 100%

For peer review only - http://bmjopen.bmj.com/site/about/guidelines.xhtml

of health benefits to be realised for both MI and stroke [31], seventeen and twenty years respectively for 50% and 100% of the health benefits to be realised for dementia, breast and colon cancer [31] and 3.2 and 8 years respectively for 50% and 100% of the health benefits to be realised for type 2 diabetes [32]. The impacts of air pollution and traffic injuries were both assumed to occur during the current year. The mode shift from car to bicycle was assumed to be the only change in exposure.

This cost-effectiveness analysis adopts the CHEERS guidelines for health economic modelling [33]. Costs are reported in euros (2017). Figure 1 shows the steps undertaken. The cost-effectiveness assumed that health care costs would be reinvested yearly and therefore discounting was not used. Costs are however also reported with a 3% discount rate.

Sensitivity analyses

Sensitivity analysis is undertaken for the same investment costs assuming that numbers of potential bicycle commuters are as few as 25% of the estimated potential bicyclists. In addition, the sensitivity to a reduced health impact and health care cost is provided. Results were reported in terms of its effect on the cost-effectiveness ratio (CER).

Estimating long term health care costs

The literature was used to inform estimates of long term health care costs for type 2 diabetes, MI, stroke, heart failure, dementia, colon cancer, breast cancer, lung cancer and asthma (Table 1). For comparability with Sweden, only papers reporting studies in high-income countries in which health care costs were met by public funds, were sought. Inclusion required that study estimates were based on administrative data recording health care costs matched to clinical diagnoses. The search was conducted in *Google Scholar*, *PubMed* and *Web of Science* between 1995 and 2018. Search terms included the health condition of interest and *lifetime/long term*

For peer review only - http://bmjopen.bmj.com/site/about/guidelines.xhtml

costs. As no studies were found that specifically reported lifetime health care costs for asthma, MI or cancer outcomes, these estimates are based on one-year average health care costs for asthma, six-year average for MI and five-year disease costs for cancer.

Estimates of the lifetime costs for bicycle related injuries were derived from a study conducted in the Netherlands which used national registry and hospital data covering the period 1998 and 2012 [34].

For peer review only

For peer review only - http://bmjopen.bmj.com/site/about/guidelines.xhtml

Table 1. Chronic disease treatment costs per case derived from the literature used to calculate health care costs

Disease	Sample size	Sample population	Data collection	Perspective	Method	Costs per case[1]	Disease duration	Reference
Type 2 Diabetes	1000	Newly diagnosed type 2 diabetes	2009-2010	Health care	Markov modelling	83 155	Life-time	Zhou et al.
Stroke	9064	All first stoke cases registered in the national register	2009	Health and societal	Aggregated costs	74 125	Life-time	Ghatnekar et. al
Dementia	111 811	Medicare beneficiaries ≥65years	1997-2005	Health care	Cohort-based simulations	13 765	Life-time	Yang et.al.
Myocardial infarction	41 210	Acute myocardial infarction incidences in Alberta, Canada	2004-2014	Health care	Cohort based aggregated costs	147 262	6-years	Tran et al.

[1] Costs per case expressed in €

For peer review only - http://bmjopen.bmj.com/site/about/guidelines.xhtml

Heart failure	1054	Olmstead county residents who fulfilled the special criteria's established for heart failure	2006	Health care	Tiangand-Huang method	113 975	Life-time	Dunlay et.al, [35]
Breast cancer	77009	Cohort of elderly Medicare, newly diagnosed cancer patients	1998-2004	Health care	5-year aggregated costs	21 177	5-years	Yabroff et.al [36]
Colon cancer	44838	Cohort of elderly Medicare, newly diagnosed cancer patients	1998-2004	Health care	5-year aggregated costs	42 446	5- years	Yabroff et.al, [36]
Lung cancer	54665	Cohort of elderly Medicare, newly diagnosed cancer patients	1998-2004	Health care	5-year aggregated costs	43 211	5-years	Yabroff et.al, [36]
Asthma	49 668	Patients with ICD-10-GM asthma code	2010	Health care	Aggregated costs	49 528 [2]	1-year	Jacob et.al,[37]

[2] Multiplied by the average length lived with the disease

For peer review only - http://bmjopen.bmj.com/site/about/guidelines.xhtml

Control group:	extracted from German sickness fund
248 340	

For peer review only

For peer review only - http://bmjopen.bmj.com/site/about/guidelines.xhtml

Results

Investment cost

The net infrastructure cost, excluding communication and maintenance costs, is €101 million. Assuming a fifty-year life cycle for the bicycle path, a €2 million annual investment gives an average annual cost of €908 per converter.

Changes in health care costs

The expected health care costs assuming the current burden of included diseases during the 50-year life span of the investment was 50 billion euros (Table 2), and 26 billion after discounting (Table 3).

The fall in chronic disease cases attributable to increased physical activity among additional bicycle commuters is estimated to save €562 million, or €241 million after discounting (3%) giving an average annual return of €11.2 million (Figure 2).

Traffic generated air pollutants were measured in particle mass concentrations [38]. Lower health care costs attributed to decreased air pollution exposure among current bicycle commuters and the general population are estimated to save €1 million annually. This is offset by increased health care costs of €0.28 million for additional bicycle commuters who are estimated to experience increased exposure to air pollution (Figure 2).

The annual additional health care costs for injuries sustained as a result of the increased accident risk among additional bicycle commuters is estimated at €1.76 million.

During the life span of the investment the estimated total health care costs for the included diseases was reduced by 1.1%. This corresponds to 8.7% of the health care expenditure that Stockholm Council planned for year 2017, or 3.7% after discounting.

For peer review only - http://bmjopen.bmj.com/site/about/guidelines.xhtml

Costs and benefits

Costs for the given scenario are compared with no assumed change in bicycle commuting. For each area of impact, the estimated change in health care costs and DALYs were calculated (Table 2). CER is calculated by subtracting investment costs from total estimated health care savings and dividing by the estimated reduction of DALYs. Based on our estimates the investment is cost-effective, averting one DALY gives a surplus of €9933.

For peer review only

For peer review only - http://bmjopen.bmj.com/site/about/guidelines.xhtml

Table 2. Estimated health care costs assuming the 2017 disease burden, changed disease burden as a result of increased bicycling, and estimated difference in health care costs

Exposure	Health care costs with the current burden of disease	Health care costs in the scenario	Estimated change in health care costs in the scenario	Percentual reduction in health care costs	Estimated change in DALYs in the scenario
Physical activity	€ 2 073 803 338	€ 1 511 162 940	€ 562 640 398	27.13	35060
Air pollution change among the general population	€ 46 283 254 008	€ 46 228 719 501	€ 54 534 507	0.12	21975
Air pollution change among additional bicyclist	€ 1 885 540 341	€ 1 899 564 512	€ - 14 024 171	-0.74	-7287
Air pollution change among current bicyclists	€ 874 825 533	€ 874 566 906	€ 258 627	0.03	131
Traffic injuries*	*	*	€ - 88 244 441	`*	-8210
Sum	€ 51 117 423 220	€ 50 602 258 300	€ 515 164 920	1.01	41669

** current costs related to traffic accidents was not available*

For peer review only - http://bmjopen.bmj.com/site/about/guidelines.xhtml

Table 3. Discounted costs assuming the 2017 disease burden, changed disease burden as a result of increased bicycling, and estimated difference in health care costs

Exposure	Health care costs with current burden of disease	Estimated change in health care costs in the scenario	Percentual reduction in health care costs
Physical activity	€ 1 092 130 168	€ 241 629 107	22.12
Air pollution change among the general population	€ 24 257 656 022	€ 25 962 558	0.11
Air pollution change among additional bicyclist	€ 988 236 242	€ - 6 687 730	-0.68
Air pollution change among current bicyclists	€ 458 507 452	€ 108 504	0.02
Traffic injuries*	*	€ - 42 117 426	*

For peer review only - http://bmjopen.bmj.com/site/about/guidelines.xhtml

Sum	€ 26 796 529 884	€ 218 895 013	0,82

** current costs related to traffic accidents was not available*

For peer review only

For peer review only - http://bmjopen.bmj.com/site/about/guidelines.xhtml

Sensitivity analysis

The scenario identified 111,487 additional bicycle commuters. The sensitivity of the estimates was assessed by assuming fewer potential bicycle commuters. If only 25% of the 111,487 potential bicycle commuters were to change their mode of transport, the CER would still result in a surplus of €2643/DALY averted (Figure 3). Even under the assumptions of a reduced health impact and lower expected reductions in health care costs, the investment remains highly cost-effective.

Discussion

This is the first health economic evaluation of this type to use an evidence-based scenario that includes information on geolocation, demography and commuting modes by origin and destination zone drawn from a population register. Investment costs were compared with estimated long term health care costs and savings attributed to increased physical activity, change in air pollution exposure and accident risk. To the best of our knowledge this is also the first economic evaluation to include morbidity health impacts in all three areas of impact. The investment is cost-effective from a health care sector perspective.

Physical activity

The largest reduction in health care costs is attributed to increased physical activity. Previous health economic assessments using hypothetical scenarios in Europe have also demonstrated cost savings in the health care sector attributed to physical activity [16 17]. In the United States (US) Grabow et al. [39] showed that, in a population of 2 million across eleven metropolitan areas, if one-half of the commutes, of less than eight kilometer round trip, were made by bicycle instead of car, mortality would be reduced by 700 premature deaths. Based on the

For peer review only - http://bmjopen.bmj.com/site/about/guidelines.xhtml

For peer review only

United States (US) statistical value of life this is equivalent to $US3.8 billion (2010 values) annually or $1900 per individual [39]. In our study the estimated cost saving attributed to reduced morbidity from increased physical activity is €562 million or €100 per annum per additional bicycle commuter.

The benefits from different types of physical acitivity are variable and not necessarily linear. In their systematic review and meta-analysis of 22 cohort studies including over 977,000 people, Woodcock et al [40] concluded that, compared with no activity, two and a half hours per week of moderate intensity activity was associated with a 19% reduction in mortality risk and seven hours per week of moderate activity reduced mortality risk by 24%. In this study physical activity effects are reported only for bicycling although adjustments were made for other likely types of physical activity based on individuals' assumed physical capacity adjusted for age and sex.

Air pollution

Some other health economic analyses have assessed the economic value of a reduction in morbidity and mortality due to a reduction in air pollution exposure. Lindsay et al [41] for example, estimated the effect of reduced air pollution exposure on mortality, numbers of active days, and acute cardiac and respiratory hospital admissions, by calculating cost per health event and the statistical value of life based on New Zealand willingness to pay studies. Grabow et al. [39] used a BenMAP cost-benefit regulatory analysis to address the potential benefits attributable to an air pollution reduction in the general population as a result of a transport mode change from car to bicycle for trips of eight kilometers or less in various US cities. Based on acute hospital costs attributable to reduced automobile exhaust and increased ozone exposure net benefits were estimated at $US 4.94 billion (2010 values).

For peer review only - http://bmjopen.bmj.com/site/about/guidelines.xhtml

To the best of our knowledge this economic evaluation is also the first to model the impact of a change in air pollution exposure within the general population as well as among current bicycle commuters. Reduced health care costs within the general population (€54 million) are offset by additional costs of €14 million among additional bicycle commuters, giving a net freed opportunity cost of €40 million within the health care budget over the 50-year life length of the investment.

Injuries

In their health economic assessment of cycling promotion in Florence, Italy, Taddei et al. [17] predicted decreased traffic accidents. Woodcock et al. [42] modelled the health impacts of active transport visions in England and Wales and also predicted a fall in road traffic and accidents under scenarios of increased walking and cycling. However using the same general scenario for increased bicycling in Stockholm County, Nilsson et al. [27] estimated health loss through increased DALYs due to increased numbers and types of traffic accidents being the first major study to highlight the negative impacts of increased injuries and fatalities resulting from the shift from car to bicycle. A strength of the Nilsson study, the results of which are incorporated into our analysis of the health impacts and costs of traffic injuries, is the use of nationally representative hospítal and police register data on injuries occured.

Head and spinal injuries costs were included in an analysis of the effects of increased active travel in urban England and Wales on the National Health Service.[16] However these estimates were four times higher than the estimates used here. The current work is based on a study by Scholten et al [34] which assumed traumatic brain injuries occurring in about 9% of bicycle related traffic accidents.

For peer review only - http://bmjopen.bmj.com/site/about/guidelines.xhtml

It has been claimed that 60% of the bicycle accidents occur due to poor infrastructure for bicycling in Sweden [43] and one of the aims of the Stockholm Bicycle Plan was to increase bicycle safety by building infrastructure for active commuters [28]. Improved road infrastructure has been shown to reduce bicycle fatalities by 45% [44] and it has been suggested that increasing the number of bicyclists can also have a "safety in numbers" effect which reduces accident risk [45]. An OECD study [46] showed that Sweden had the world's safest roads with the lowest number of fatal accidents per billion-cycled kilometers compared with other European countries.

Total economic benefits

Health care costs

This study shows a net benefit corresponding to in total 8.7% of the 2017 Stockholm County health care budget after 50 years. Grabow et al. [39] reported a 5% reduction in health care cost attributable to a change in air pollution exposure and increased physical activity. In a scenario of daily bicycling of 3.4 kilometers among urban residents in England and Wales, Jarrett et al. [16] reported that the costs for England National Health Service would be reduced by 0.8% as a consequence of increased physical activity.

Economic assessment

It is assumed that investment in physical infrastructure would lead to increased physical activity levels, and flow-on cost reductions within the health care sector. These assumptions have been adopted by others undertaking similar economic evaluations in which investment in infrastructure to promote active transport is assumed to increase physical activity and reduce air pollution. For example, a study conducted in Dane County in the US reported a cost benefit ratio of 1.87 over a ten-year cycle attributed to a $US450 million investment in

For peer review only - http://bmjopen.bmj.com/site/about/guidelines.xhtml

sidewalks [47]. Some researchers suggest that the saved opportunity costs could finance the investment. For example a study of air quality and exercise-related health benefits resulting from reduced car travel in the Midwestern US Grabow et al.[39] concluded that the estimated reduction in health care costs could finance the infrastructure change within one to ten years. In Italy Taddei et al. [17] estimated that health care costs saved due to a reduction in NCDs could cover 50 kilometers of new bicycle roads within seven to ten years in Florence.

Strengths and limitations

Strengths

Many previous health economic analyses of active transport interventions have used hypothetical or proposed active travel interventions, however the scenario in this study was constructed linking register data that included home and work address coordinates [25]. Gender and age-specific expected bicycle speeds were used to assess individuals' capacity to commute by bicycle between home and work in 30 minutes or less [22 25]. In their systematic review of active transport interventions that include physical activity benefits, Brown et al [48] reported a limited inclusion of health effects with few studies reporting reductions in morbidity as well as mortality. Morbidity reduction is captured in the DALY weights in this study and the impacts of mortality under this scenario have been estimated in a companion paper [26]. Additionally, the sensitivity analysis showed that the intervention is highly cost-effective even if only one quarter of the potential bicycle commuters were to take up bicycle commuting.

Limitations

We acknowledge the limitations in the health economic modelling. It is fair to assume that improvements in population health would still occur without the intervention or increased bicycling. However, modelling all possible behavioural changes under uncertainty and

For peer review only - http://bmjopen.bmj.com/site/about/guidelines.xhtml

generalising these to population health outcomes in the coming fifty years would be a complex exercise beyond the scope of this study. Furthermore, it would not be meaningful to base predictions on possible future health trends for which there is a lack of reliable data. Such is the case regarding air pollution trends in Stockholm since 2010 [49]. Cost-effectiveness studies facing these and similar limitations have been published previously [50].

The health impact estimates our economic assessment are based on the Global Burden of Disease [51]. We have not included all possible health impacts. We did not, for example, account for impacts on mental health (e.g. depression) and obesity that are expected to result from increased physical activity [52 53]. A study in the US reported that obesity reductions resulting from active transport could save $90.93 million in health care costs [12].

It is possible that health benefits and cost savings are underestimated in this study due to not fully accounting for the benefits of reduced obesity and improved well-being that may accrue over time and flow-on to the broader community. These effects would be considered in an economic evaluation conducted from a societal perspective, which is beyond the scope of the present analysis. There are other potential savings which were not included. For example, it has been shown that people value the time bicycling to work more than the time spent in motorized transport [54]. If a societal perspective had been applied cost-effectiveness may have been higher.

Except for the effect of the mode-shift towards increased bicycling, no other changes in air pollution concentrations were assumed. In terms of air pollution effects, the US Environmental Protection Agency applies segmented lag-times for mortality estimates [55]. However, due to the lack of studies on lag times for air pollution effects on morbidity, it was assumed that effects occurred in the same year as exposure.

For peer review only

For peer review only - http://bmjopen.bmj.com/site/about/guidelines.xhtml

In using secondary data to estimate long term costs for chronic NCDs there was a lack of evidence for cancer and asthma patients. Estimates are therefore based on best approximations from scientific evidence published in the peer-reviewed literature. Although it is common practice for economic evaluations to draw upon evidence from previous studies, comparing evidence from studies with different methodological approaches in different settings is not without its shortcomings [56]. Studies from other high-income countries were used so that the results could be reasonably generalised to Sweden.

Conclusions

From a health care perspective investing in urban infrastructure to increase bicycling in Stockholm is cost-effective in relation to the Swedish threshold value of € 53,000 per DALY averted. This evaluation makes best use of available data and calculates estimates based on a previously developed realistic scenario. More work of this type is needed in order to build an evidence base for decision-makers responsible for investing funds in public infrastructure.

Author Contributions

HKK planned the health economic study and undertook the data collection and economic analysis with input from LL. HKK wrote the first draft. JSW reviewed the overall content, provided input and revised and re-drafted the paper in conjunction with HKK. BF advised on the planning and conduct of the study and was responsible for the overall transport scenario project of which this work was part. JNS provided health impact estimates and critical input into the planning and conduct of the study, assisted with the statistical analyses, and helped revise the drafts. All authors read and approved the final version of the manuscript.

For peer review only - http://bmjopen.bmj.com/site/about/guidelines.xhtml

For peer review only

Role of the funding source

This work was funded by the Swedish Research Council for Health, Working Life and Welfare (grant number 2012-1296). The paper reflect only the authors views, and the funder had no role in the design or conduct of the study.

Competing interests

All authors have completed the ICMJE uniform disclosure form at www.icmje.org/coi_disclosure.pdf and declare: no support from any organization for the submitted work; no financial relationships with any organizations that might have an interest in the submitted work in the previous three years; no other relationships or activities that could appear to have influenced the submitted work.

Transparency declaration

The lead author affirms that the manuscript is an honest, accurate, and transparent account of the study being reported; that no important aspects of the study have been omitted; and that any discrepancies from the study as planned have been explained.

Patient and Public involvement: No patient or public involvement.

The datasets analyzed during the current study are available from the corresponding author on reasonable request.

Acknowledgements

Bertil Forsberg received a grant (2012-1296) from FORTE (Forskningsrådet för hälsa, arbetsliv och välfärd) for the transport scenario study. We thank study leaders and co-authors in the transport scenario study, in particular Peter Schantz, Christer Johansson, Wasif Raza and Magnus Strömgren.

For peer review only - http://bmjopen.bmj.com/site/about/guidelines.xhtml

We are grateful to the reviewers who have challenged us with constructive comments and excellent scientific feedback.

Figure legend/caption

Figure 1. Flowchart for estimated chain of events as the base for conducting the cost-effectiveness analysis

Figure 2. Estimated yearly expenditure averted (in millions) in the health care sector due to increased physical activity, change in air pollution concentrations and risk of traffic injuries

Figure 3. Sensitivity analysis of the CER considering number of obtained bicyclists, the health impact, and health care costs

Data sharing statement: No additional data are available. Health care and investment costs data reported in the study and health outcomes are reported in Sommar et al. 2019 [25]

For peer review only

For peer review only - http://bmjopen.bmj.com/site/about/guidelines.xhtml

References

1. World Health Organization. 2008-2013 Action Plan for the Global Strategy for the Prevention and Control of Noncommunicable Diseases. Genève, 2008:48.

2. Cecchini M, Sassi F, Laver JA, et al. Tackling of unhealthy diets, physical inactivity, and obesity: health effects and cost-effectiveness. *The Lancet* 2010;376:1775-84. doi: DOI:10.1016/S0140-6736(10)61514-0

3. World Health Organization. Global recommendations on physical activity for health. Genève, 2010:60.

4. Lee I-M, Shiroma EJ, Lobelo F, et al. Effect of physical inactivity on major non-communicable diseases worldwide: an analysis of burden of disease and life expectancy. *The Lancet* 2012;380:219-29. doi: http://dx.doi.org/10.1016/50140-6736(12)61031-9

5. World Health Organization, OECD, European Observatory. Promoting health, preventing disease. The economic case. In: Davis McDaid FS, Sherry Merkur, ed. New York, 2015:370.

6. World Health Organization. Physical Activity Fact Sheet. 2016

7. Cecchini M, Bull F. Promoting physical activity. In: David McDaid FS, Sherry Merkur, ed. Promoting health, preventing disease The economic case. New York: McGraw Hill Education 2015:101-34.

8. World Health Organization. Urbanisation and Health. *Bulletin of the World Health Organization* 2010;88:245-46.

9. United Nations. Sustainable Development Goals. 2015

10. IPCC. Climate change 2007 - Mitigation of climate change. 2007

11. World Health Organization Regional Office for Europe, OECD. Economic cost of the health impact of air pollution in Europe: clean air, health and wealth. Copenhagen, 2015:66.

12. Pucher J, Buehler R, Bassett DR, et al. Walking and cycling to health: a comparative analysis of city, state, and international data. *Am J Public Health* 2010;100(10):1986-92. doi: 10.2105/ajph.2009.189324 [published Online First: 2010/08/21]

13. Fraser SD, Lock K. Cycling for transport and public health: a systematic review of the effect of the environment on cycling. *Eur J Public Health* 2011;21(6):738-43. doi: 10.1093/eurpub/ckq145 [published Online First: 2010/10/12]

14. Cobiac LJ, Vos T, Barendregt JJ. Cost-effectiveness of interventions to promote physical activity: a modelling study. *PLoS Med* 2009;6(7):e1000110. doi: 10.1371/journal.pmed.1000110 [published Online First: 2009/07/15]

15. Mulley C, Tyson R, McCue P, et al. Valuing active travel: including the health benefits of sustainable transport in transportation appraisal frameworks. *Research in Transportation Business and Management* 2013;7:27-34.

16. Jarrett J, Woodcock J, Griffiths UK, et al. Effect of increasing active travel in urban England and

For peer review only - http://bmjopen.bmj.com/site/about/guidelines.xhtml

Wales on costs to the National Health Service. *The Lancet* 2012;379(9832):2198-205. doi: 10.1016/s0140-6736(12)60766-1 [published Online First: 2012/06/12]

17. Taddei C, Gnesotto R, Forni S, et al. Cycling promotion and non-communicable disease prevention: health impact assessment and economic evaluation of cycling to work or school in Florence. *PLoS One* 2015;10(4):e0125491. doi: 10.1371/journal.pone.0125491 [published Online First: 2015/05/01]

18. Beale SJ, Bending MW, Trueman P, et al. Should we invest in environmental interventions to encourage physical activity in England? An economic appraisal. *Eur J Public Health* 2012;22(6):869-73. doi: https://doi.org/10.1093/eurpub/ckr151

19. Hanson S, Jones A. Missed opportunities in the evaluation of public health interventions: a case study of physical activity programmes. *BMC Public Health* 2017;17(1):674. doi: 10.1186/s12889-017-4683-z [published Online First: 2017/08/24]

20. Weatherly H, Drummond M, Claxton K, et al. Methods for assessing the cost-effectiveness of public health interventions: key challenges and recommendations. *Health Policy* 2009;93(2-3):85-92. doi: 10.1016/j.healthpol.2009.07.012 [published Online First: 2009/08/28]

21. OECD/EU. Health at a Glance: Europe 2016 - State of Health in the EU Cycle. Paris, 2016:204.

22. Strömgren M, Schantz. P, Nilsson Sommar J, et al. Modelling a commuter modal shift from car trips to cycling: Scenario construction and outcomes for Stockholm County, Sweden. To be submitted., 2019.

23. Stjenström O. The Astrid data base and population geography (only in Swedish "Databasen ASTRID och befolkningsgeografi exemplen integration och barnfamiljenas geografi" *Geografiska Notised*;60. No.2

24. Jonsson D BS, Almström P, Algers S. The usefulness of transport models in Swedish planning practice. *Transport Reviews* 2011;31(2):251-65.

25. Nilsson Sommar J, Schantz P, Strömgren M, et al. The potential for reduced mortality by increased cycle commuting - a health impact assessment using register data on home and work addresses in Stockholm, Sweden. To be submitted., 2019.

26. Nilsson Sommar J, Johansson C, Lövenheim B, et al. Overall health impacts of a potential increase in active commuting in Stockholm, Sweden. To be submitted., 2019.

27. Nilsson P, Stigson H, Ohlin M, et al. Modelling the effect on injuries and fatalities when changing mode of transport from car to bicycle. *Accid Anal Prev* 2017;100:30-36. doi: 10.1016/j.aap.2016.12.020 [published Online First: 2017/01/14]

28. En el av Framkomlighetsstrategin: Cyckelplan. In: Stad S, ed., 2016.

29. Livslängs och Trafikanalys. In: Trafikverket, ed., 2002:6.

30. Stockholm Läns Lansting Hälsa- och sjukvårdsnämden. Slutlig budget 2017 HSN: Förändringsarbete i fokus när vården i länet växer, 2017.

For peer review only

For peer review only - http://bmjopen.bmj.com/site/about/guidelines.xhtml

31. Kahlmeier S CN DH, et al. . Health economic assessment tools (HEAT) for walking and cycling: methodology and user guide. *Copenhagen: WHO Regional Office Europe* 2011

32. Umpierre D, Ribeiro PA, Kramer CK, et al. Physical activity advice only or structured exercise training and association with HbA1c levels in type 2 diabetes: a systematic review and meta-analysis. *JAMA* 2011;305(17):1790-9. doi: 10.1001/jama.2011.576 [published Online First: 2011/05/05]

33. Husereau D, Drummond M, Petrou S, et al. Consolidated Health Economic Evaluation Reporting Standards (CHEERS)--explanation and elaboration: a report of the ISPOR Health Economic Evaluation Publication Guidelines Good Reporting Practices Task Force. *Value Health* 2013;16(2):231-50. doi: 10.1016/j.jval.2013.02.002 [published Online First: 2013/03/30]

34. Scholten AC, Polinder S, Panneman MJ, et al. Incidence and costs of bicycle-related traumatic brain injuries in the Netherlands. *Accid Anal Prev* 2015;81:51-60. doi: 10.1016/j.aap.2015.04.022 [published Online First: 2015/05/06]

35. Dunlay SM, Shah ND, Shi Q, et al. Lifetime costs of medical care after heart failure diagnosis. *Circ Cardiovasc Qual Outcomes* 2011;4(1):68-75. doi: 10.1161/circoutcomes.110.957225 [published Online First: 2010/12/09]

36. Yabroff KR, Lamont EB, Mariotto A, et al. Cost of care for elderly cancer patients in the United States. *J Natl Cancer Inst* 2008;100(9):630-41. doi: 10.1093/jnci/djn103 [published Online First: 2008/05/01]

37. Jacob C, Bechtel B, Engel S, et al. Healthcare costs and resource utilization of asthma in Germany: a claims data analysis. *Eur J Health Econ* 2016;17(2):195-201. doi: 10.1007/s10198-015-0671-3 [published Online First: 2015/02/27]

38. Johansson C, Lovenheim B, Schantz P, et al. Impacts on air pollution and health by changing commuting from car to bicycle. *Sci Total Environ* 2017;584-585:55-63. doi: 10.1016/j.scitotenv.2017.01.145 [published Online First: 2017/01/31]

39. Grabow ML, Spak SN, Holloway T, et al. Air quality and exercise-related health benefits from reduced car travel in the midwestern United States. *Environ Health Perspect* 2012;120(1):68-76. doi: 10.1289/ehp.1103440 [published Online First: 2011/11/04]

40. Woodcock J, Franco OH, Orsini N, et al. Non-vigorous physical activity and all-cause mortality: systematic review and meta-analysis of cohort studies. *Int J Epidemiology* 2011;40(1):121-38. doi: 10.1093/ije/dyq104 [published Online First: 2010/07/16]

41. Lindsay G, Macmillan A, Woodward A. Moving urban trips from cars to bicycles: impact on health and emissions. *Australian and New Zealand Journal of Public Health* 2011;35(1):54-60. doi: 10.1111/j.1753-6405.2010.00621.x [published Online First: 2011/02/09]

42. Woodcock J, Givoni M, Morgan AS. Health impact modelling of active travel visions for England and Wales using an Integrated Transport and Health Impact Modelling Tool (ITHIM). *PLoS One* 2013;8(1):e51462. doi: 10.1371/journal.pone.0051462 [published Online First: 2013/01/18]

43. Niska A G, S, Nyberg, J, Eriksson, J. . Cyklisters singelolyckor - Analys av olycks- och skadedata samt djupintervjuer (sIngle bicycle accidents analysis of hospital injury data and interviews). *VTI, Linköping, Sweden* 2013

For peer review only

For peer review only - http://bmjopen.bmj.com/site/about/guidelines.xhtml

44. Schepers PM, Rob; Wegman, Fred; Heinen, Eva, . Road safety and bicycle usage impacts of unbundling vehicular and cycle traffic in Dutch urban networks. *European Journal of Transport and Infrastructure Research* 2013;13(3):221-38.

45. Jacobsen PL. Safety in numbers: more walkers and bicyclists, safer walking and bicycling. *Inj Prev* 2015;21(4):271-5. doi: 10.1136/ip.9.3.205rep [published Online First: 2015/07/24]

46. OECD/EU. Road Safety Annual Report 2011. OECD International Transport Forum, Paris, 2012.

47. Guo JY, Gandavarapu S. An economic evaluation of health-promotive built environment changes. *Prev Med* 2010;50 Suppl 1:S44-9. doi: 10.1016/j.ypmed.2009.08.019 [published Online First: 2009/10/21]

48. Brown V, Diomedi, B., Moodie, M., Veerman, J., & Carter, R. A systematic review of economic analyses of active transport interventions that include physical activity benefits. *Transport Policy* 2016;45(C):190-208. doi: 10.1016/j.tranpol.2015.10.003

49. Olstrup H, Forsberg B, Orru H, et al. Trends in air pollutants and health impacts in three Swedish cities over the past three decades. *Atmospheric Chemistry and Physics* 2018;18(21):15705–23. doi: https://doi.org/10.5194/acp-18-15705-2018

50. Lindholm L, Stenling A, Norberg M, et al. A cost-effectiveness analysis of a community based CVD program in Sweden based on a retrospective register cohort. , 18(1), 1–7. *BMC Public Health* 2018;18(1):1-7. doi: https://doi.org/10.1186/s12889-018-5339-3

51. World Health Organization. Global burden of disease 2004 update: disability weights for diseases and conditions. Genève: WHO, 2004:9.

52. Gill JM, Malkova D. Physical activity, fitness and cardiovascular disease risk in adults: interactions with insulin resistance and obesity. *Clinical Science (London, England : 1979)* 2006;110(4):409-25. doi: 10.1042/cs20050207 [published Online First: 2006/03/11]

53. Warburton DE, Nicol CW, Bredin SS. Health benefits of physical activity: the evidence. *CMAJ : Canadian Medical Association Journal = journal de l'Association medicale canadienne* 2006;174(6):801-9. doi: 10.1503/cmaj.051351 [published Online First: 2006/03/15]

54. Borjesson M EJ. The value of time and external benefits in bicycle appraisal. *Transport Research Part A Policy and Practice* 2012;46(4):673-83.

55. U.S. Environmental Protection Agency Office of Air Quality Planning and Standards Health and Environmental Impacts Division. Regulatory Impact Analysis for the Final Revisions to the National Ambient Air Quality Standards for Particulate Matter. EPA-452/R-12-005. Research Triangle Park, NC 27711, 2012:474.

56. Briggs A CC, Sculpher M. Decision Modelling for Health Economic Evaluation. Oxford University Press, 2011.

For peer review only - http://bmjopen.bmj.com/site/about/guidelines.xhtml

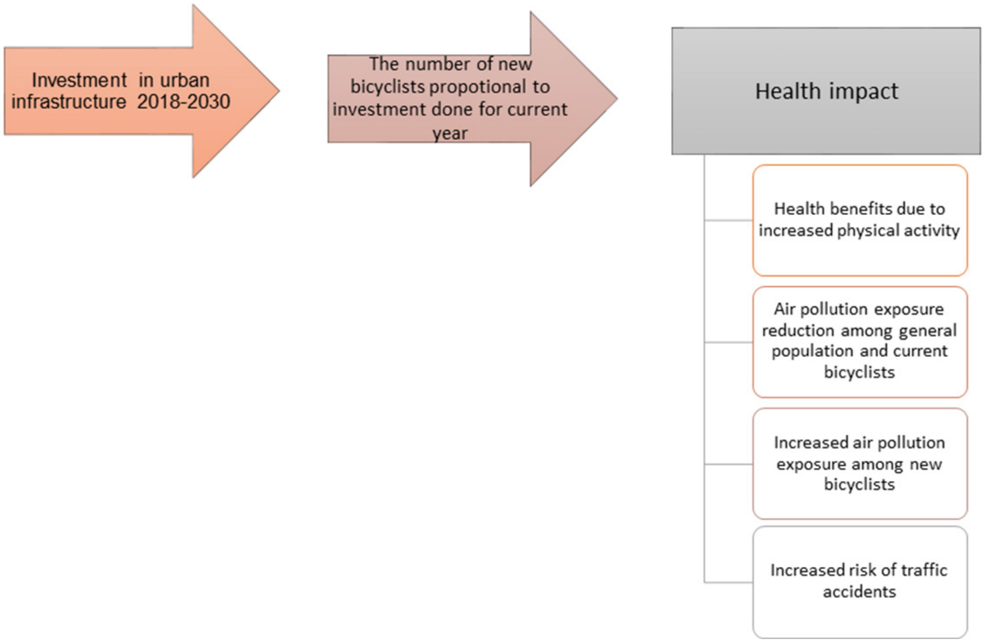

Figure 1. Flowchart for estimated chain of events as the base for conducting the cost-effectivness analysis

96x66mm (300 x 300 DPI)

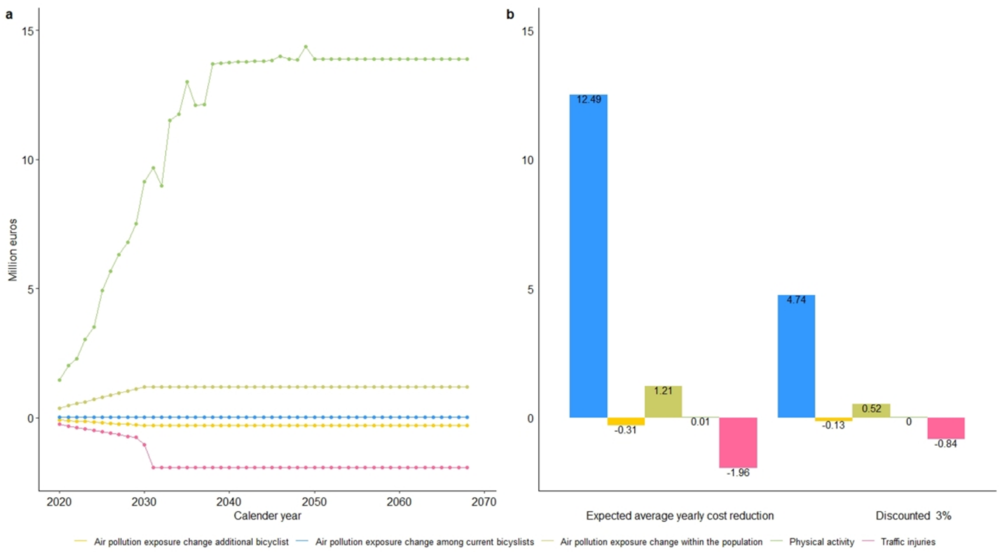

Figure 2. Estimated yearly expenditure averted in the health care sector due to increased physical activity, change in air pollution concentrations and risk of traffic injuries

383x211mm (300 x 300 DPI)

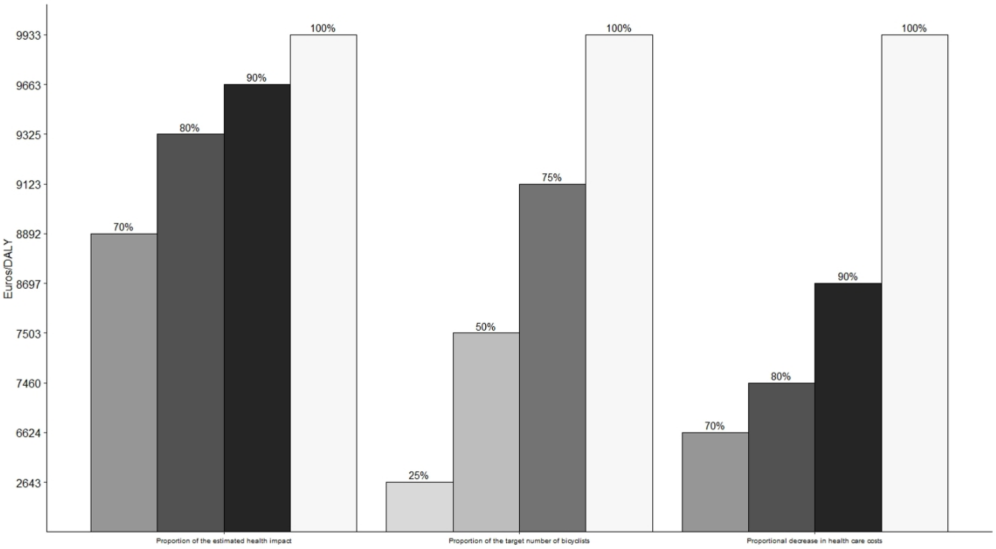

Figure 3. Sensitivity analysis of the CER considering number of obtained bicyclists, the health impact, and health care costs

383x211mm (300 x 300 DPI)

CHEERS Checklist
Items to include when reporting economic evaluations of health interventions

The **ISPOR CHEERS Task Force Report**, *Consolidated Health Economic Evaluation Reporting Standards (CHEERS)—Explanation and Elaboration: A Report of the ISPOR Health Economic Evaluations Publication Guidelines Good Reporting Practices Task Force*, provides examples and further discussion of the 24-item CHEERS Checklist and the CHEERS Statement. It may be accessed via the *Value in Health* or via the ISPOR Health Economic Evaluation Publication Guidelines – CHEERS: Good Reporting Practices webpage: http://www.ispor.org/TaskForces/EconomicPubGuidelines.asp

Section/item	Item No	Recommendation	Reported on page No/ line No
**Title and abstract**			
Title	1	Identify the study as an economic evaluation or use more specific terms such as “cost-effectiveness analysis”, and describe the interventions compared.	1/1-2
Abstract	2	Provide a structured summary of objectives, perspective, setting, methods (including study design and inputs), results (including base case and uncertainty analyses), and conclusions.	2-3/25
**Introduction**			
Background and objectives	3	Provide an explicit statement of the broader context for the study. Present the study question and its relevance for health policy or practice decisions.	5/107-110
**Methods**			
Target population and subgroups	4	Describe characteristics of the base case population and subgroups analysed, including why they were chosen.	6/115-116
Setting and location	5	State relevant aspects of the system(s) in which the decision(s) need(s) to be made.	NA
Study perspective	6	Describe the perspective of the study and relate this to the costs being evaluated.	6/117
Comparators	7	Describe the interventions or strategies being compared and state why they were chosen.	6/124-126
Time horizon	8	State the time horizon(s) over which costs and consequences are being evaluated and say why appropriate.	6/122-124
Discount rate	9	Report the choice of discount rate(s) used for costs and outcomes and say why appropriate.	9/185-187
Choice of health outcomes	10	Describe what outcomes were used as the measure(s) of benefit in the evaluation and their relevance for the type of analysis performed.	7/144-151
Measurement of effectiveness	11a	*Single study-based estimates:* Describe fully the design features of the single effectiveness study and why the single study was a sufficient source of clinical effectiveness data.	NA

For peer review only

	11b	*Synthesis-based estimates:* Describe fully the methods used for identification of included studies and synthesis of clinical effectiveness data.	9/194
Measurement and valuation of preference based outcomes	12	If applicable, describe the population and methods used to elicit preferences for outcomes.	NA
Estimating resources and costs	13a	*Single study-based economic evaluation:* Describe approaches used to estimate resource use associated with the alternative interventions. Describe primary or secondary research methods for valuing each resource item in terms of its unit cost. Describe any adjustments made to approximate to opportunity costs.	NA
	13b	*Model-based economic evaluation:* Describe approaches and data sources used to estimate resource use associated with model health states. Describe primary or secondary research methods for valuing each resource item in terms of its unit cost. Describe any adjustments made to approximate to opportunity costs.	10/201-206
Currency, price date, and conversion	14	Report the dates of the estimated resource quantities and unit costs. Describe methods for adjusting estimated unit costs to the year of reported costs if necessary. Describe methods for converting costs into a common currency base and the exchange rate.	9/184
Choice of model	15	Describe and give reasons for the specific type of decision-analytical model used. Providing a figure to show model structure is strongly recommended.	8/158 Figure 1
Assumptions	16	Describe all structural or other assumptions underpinning the decision-analytical model.	8/169-174
Analytical methods	17	Describe all analytical methods supporting the evaluation. This could include methods for dealing with skewed, missing, or censored data; extrapolation methods; methods for pooling data; approaches to validate or make adjustments (such as half cycle corrections) to a model; and methods for handling population heterogeneity and uncertainty.	9/189
**Results**			
Study parameters	18	Report the values, ranges, references, and, if used, probability distributions for all parameters. Report reasons or sources for distributions used to represent uncertainty where appropriate. Providing a table to show the input values is strongly recommended.	11-13/Tabel 1
Incremental costs and outcomes	19	For each intervention, report mean values for the main categories of estimated costs and outcomes of interest, as well as mean differences between the comparator groups. If applicable, report incremental cost-effectiveness ratios.	16/231 Tabel 2
Characterising uncertainty	20a	*Single study-based economic evaluation:* Describe the effects of sampling uncertainty for the estimated incremental cost and incremental effectiveness parameters, together with the impact	NA

For peer review only

		of methodological assumptions (such as discount rate, study perspective).	
	20b	*Model-based economic evaluation:* Describe the effects on the results of uncertainty for all input parameters, and uncertainty related to the structure of the model and assumptions.	19/264
Characterising heterogeneity	21	If applicable, report differences in costs, outcomes, or cost-effectiveness that can be explained by variations between subgroups of patients with different baseline characteristics or other observed variability in effects that are not reducible by more information.	NA
**Discussion**			
Study findings, limitations, generalisability, and current knowledge	22	Summarise key study findings and describe how they support the conclusions reached. Discuss limitations and the generalisability of the findings and how the findings fit with current knowledge.	19/270
**Other**			
Source of funding	23	Describe how the study was funded and the role of the funder in the identification, design, conduct, and reporting of the analysis. Describe other non-monetary sources of support.	26/420-429
Conflicts of interest	24	Describe any potential for conflict of interest of study contributors in accordance with journal policy. In the absence of a journal policy, we recommend authors comply with International Committee of Medical Journal Editors recommendations.	

For consistency, the CHEERS Statement checklist format is based on the format of the CONSORT statement checklist

The **ISPOR CHEERS Task Force Report** provides examples and further discussion of the 24-item CHEERS Checklist and the CHEERS Statement. It may be accessed via the *Value in Health* link or via the ISPOR Health Economic Evaluation Publication Guidelines – CHEERS: Good Reporting Practices webpage: http://www.ispor.org/TaskForces/EconomicPubGuidelines.asp

The citation for the CHEERS Task Force Report is:

Husereau D, Drummond M, Petrou S, et al. Consolidated health economic evaluation reporting standards (CHEERS)—Explanation and elaboration: A report of the ISPOR health economic evaluations publication guidelines good reporting practices task force. Value Health 2013;16:231-50.

For peer review only
